# Upf3a but not Upf1 mediates the genetic compensation response induced by *leg1* deleterious mutations in an H3K4me3-independent manner

**DOI:** 10.1038/s41421-023-00550-2

**Published:** 2023-06-27

**Authors:** Aixuan Xie, Zhipeng Ma, Jinyang Wang, Yuxi Zhang, Yayue Chen, Chun Yang, Jun Chen, Jinrong Peng

**Affiliations:** 1 1MOE Key Laboratory of Biosystems Homeostasis & Protection, College of Animal Sciences, Zhejiang University, Hangzhou, Zhejiang China; 2 College of Life Sciences, Zhejiang University, Hangzhou, Zhejiang China

**Keywords:** Developmental biology, Transcription

## Abstract

Genetic compensation responses (GCRs) can be induced by deleterious mutations in living organisms in order to maintain genetic robustness. One type of GCRs, homology-dependent GCR (HDGCR), involves transcriptional activation of one or more homologous genes related to the mutated gene. In zebrafish, ~80% of the genetic mutants produced by gene editing technology failed to show obvious phenotypes. The HDGCR has been proposed to be one of the main reasons for this phenomenon. It is triggered by mutant mRNA bearing a premature termination codon and has been suggested to depend on components of both the nonsense mRNA-mediated degradation (NMD) pathway and the complex of proteins associated with Set1 (COMPASS). However, exactly which specific NMD factor is required for HDGCR remains disputed. Here, zebrafish *leg1* deleterious mutants are adopted as a model to distinguish the role of the NMD factors Upf1 and Upf3a in HDGCR. Four single mutant lines and three double mutant lines were produced. The RNA-seq data from 71 samples and the ULI-NChIP-seq data from 8 samples were then analyzed to study the HDGCR in *leg1* mutants. Our results provide strong evidence that Upf3a, but not Upf1, is essential for the HDGCR induced by nonsense mutations in *leg1* genes where H3K4me3 enrichment appears not to be a prerequisite. We also show that Upf3a is responsible for correcting the expression of hundreds of genes that would otherwise be dysregulated in the *leg1* deleterious mutant.

## Introduction

Living organisms have developed a variety of means to cope with environmental changes and genetic variations for their viability and fitness during evolution^1^. The genetic compensation responses (GCRs), including the use of redundant genes, genetic network rewiring, alternative splicing, genetic modifiers, redundant signaling pathways, and other similar processes and mechanisms have been widely adopted and are evident across the broad range of the biological taxa^2^. For example, regarding genetic network rewiring, by building a protein–protein interaction network containing 1870 proteins in the yeast *Saccharomyces cerevisiae* (*S. cerevisiae*), Jeong et al. found a close correlation between the lethality of a single-gene deletion with the topological position of its protein product in the web of molecular interactions^3^. As for genetic modifiers, through examining survival assays of ~5000 unique single-gene deletions in *S. cerevisiae*, Teng et al. found that most of these gene deletion strains had one additional corresponding mutant gene which appeared to be responsible for adaptive genetic changes^4^. Although the importance of the GCR for maintaining genetic robustness is generally appreciated, the field is young and the different classes of GCRs have not yet been fully fleshed out. The homology-dependent GCR (HDGCR) is an important subset of GCR, mainly due to the high abundance of homologous genes in the genome, which provides accessible resources for functional compensation. For example, the percentage of homologous genes corresponding to coding genes is approximately 65%, 63%, 72%, and 78% for the genome of *Homo sapiens*^5,6^, *Mus musculus*^7^, *Arabidopsis thaliana*^8^ and *Danio rerio* (zebrafish)^9^, respectively. This neatly fits the observation that ~80% of the genetic mutants produced by gene editing technology fail to show an obvious phenotype in zebrafish^10^. HDGCRs induced by deleterious mutations are therefore suggested to account for this phenomenon^11^. Recently, two reports have demonstrated that the HDGCR relies on: (1) mutant mRNA bearing a premature termination codon (PTC); (2) a homologous sequence occurring between the mutated gene and its compensatory homologs; (3) components in the nonsense mRNA-mediated degradation pathway (NMD pathway); and (4) promotion of H3K4me3 modification at the transcription start site (TSS) of the compensated genes via the complex of proteins associated with Set1 (COMPASS)^12,13^. A key biological function of the NMD pathway is to cleanse the abnormal transcripts carrying a PTC^14^, while the COMPASS is responsible for the H3K4 methylation around the TSS site^15,16^. Upf1 and Upf3b have been shown to be key positive regulators of the NMD pathway whereas Upf3a, a homolog of Upf3b, appears to play a minor or even antagonistic role in NMD pathway^14,17,18^. The two previous related reports, one from our lab and the other from Stainier’s lab, have put forward two differing proposals for the factor(s) responsible for relaying the HDGCR signal from the NMD complex to the COMPASS complex. The report from Stainier’s lab proposed that Upf1 and mutant mRNA degradation are required for the activation of the HDGCR^12^, whereas we showed that HDGCR depends on Upf3a^13^. Based on this discrepancy, there is a need to clarify the role of Upf1 and Upf3a in the process of the HDGCR.

Liver-enriched gene 1 (Leg1) represents a protein family highly conserved in vertebrates. It is characterized by a single domain of unknown function 781 (DUF781)^19–22^. In zebrafish, Leg1 is a liver-produced novel serum protein^20^, whereas, in the platypus and echidna, the Leg1 family member known as Monotreme Lactation Protein (MLP) is a component of milk that may function as an antimicrobial protein^23,24^. The zebrafish genome contains two copies of *leg1* genes, *leg1a* and *leg1b*, which are closely linked on chromosome 20^20^. Both *leg1a* and *leg1b* transcripts are detectable at the embryonic stage, but with *leg1a* being the predominant form (> 90%) at this stage of development^20,21^. We previously showed that knockdown using gene-specific morpholinos (MOs) of either *leg1a*, *leg1b*, or both together, resulted in a small liver at 3.5 days post-fertilization (dpf)^20^. In contrast, except for during the winter season, knockout of the *leg1a* gene (the *leg1a*^*zju1/zju1*^ mutant) did not affect liver development. This was likely due to an upregulation of *leg1b* expression discernable from 3 dpf onwards^21^, suggesting the activation of an HDGCR in the *leg1a*^*zju1/zju1*^ mutant embryos.

In this report, using RNA sequencing (RNA-seq) we studied *leg1b* expressions in *leg1a* mutant embryos and distinguished the role of Upf1 and Upf3a in the HDGCR by analyzing *leg1b* expression in the *leg1a*^*zju1/zju1*^*upf3a*^*−/−*^ double and *leg1a*^*zju1/zju1*^*upf1*^*−/−*^ double mutants at either 3 dpf or 5 dpf. We also performed ultra-low-input micrococcal nuclease-based native chromatin immunoprecipitation and sequencing (ULI-NChIP-seq) analysis using the micro-dissected liver buds at 5 dpf to study the correlation between the expression of 70 liver-enriched genes and H3K4me3 modification. Our data show that Upf3a, but not Upf1, is crucial for the HDGCR in *leg1a* mutants at 3 dpf and 5 dpf.

## Results

### Only *leg1a* and *leg1b* double mutations confer a small liver phenotype

**Fig. 1:**
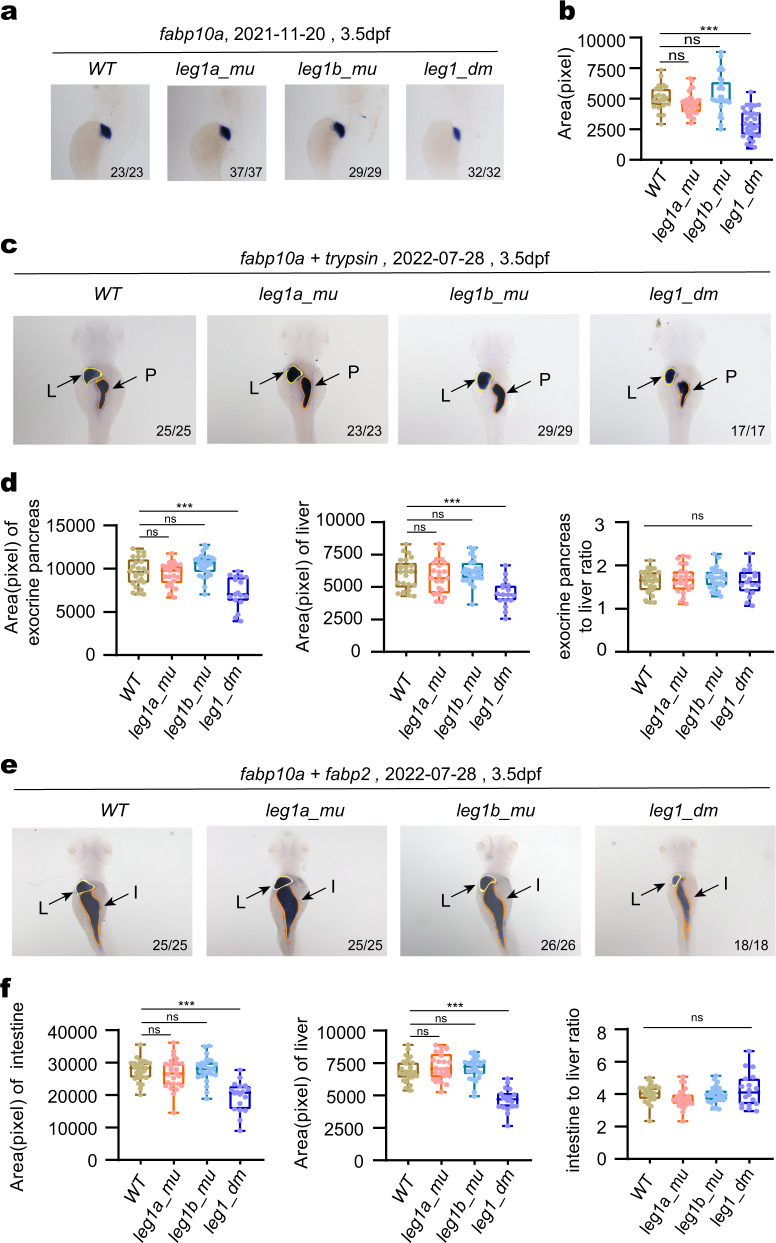
*leg1a*^*zju3/zju3*^*leg1b*^*zju1/zju1*^ double mutant exhibits a smaller liver and exocrine pancreas and thinner intestinal tube phenotype. **a**, **b** Representative WISH images using the *fabp10a* probe (liver marker) at 3.5 dpf (**a**) and statistical analysis of the liver size (**b**). *leg1a*^*zju3/zju3*^*leg1b*^*zju1/zju1*^ double homozygous mutants (*leg1_dm*) exhibited a small liver phenotype when compared with WT, *leg1a*^*zju1/zju1*^ (*leg1a_mu*) single and *leg1b*^*zju1/zju1*^ (*leg1b_mu*) single homozygous mutants. **a** Top: the date of experiments performed; bottom right: number of embryos showing the phenotype over total embryos examined. **b** The *y* axis shows the positive signal area in each embryo stained with the *fabp10a* probe. **c**–**f** Representative WISH images using the combination of *fabp10a* (liver) and *trypsin* (exocrine pancreas) (**c**) and of *fabp10a* (liver) and *fabp2* (intestine marker) (**e**) probes on WT, *leg1a_mu* single, *leg1b_mu* single and *leg1_dm* double mutant embryos at 3.5 dpf. **c**, **e** Top: the date of experiments performed; bottom right: number of embryos showing the phenotype over total embryos examined. L, liver (outlined by a yellow line in **c**, **e**); P, exocrine pancreas (outlined by a magenta line in **c**); I, intestinal tube (outlined by a magenta line in **e**). Statistical analysis of the sizes of exocrine pancreas (left) and liver (middle) (**d**) and of the intestine (left) and liver (middle) (**f**) was performed, respectively. The ratios of liver vs exocrine pancreas (right panel in **d**) and of liver vs intestine (right panel in **f**) for each genotype were also shown. ***P < 0.001; ns, no significance.

To find out whether the HDGCR is activated in the *leg1a* or *leg1b* deleterious mutants, we took the advantage of the PTC-bearing *leg1a*^*zju1*^ single (with a 13 bp insertion in the exon 1 of *leg1a*), *leg1b*^*zju1*^ single (with a 14 bp deletion in the exon 2 of *leg1b*) and *leg1a*^*zju3*^*leg1b*^*zju1*^ double (where *leg1a*^*zju3*^ harbors a 7 bp deletion in *leg1a*) mutant lines available to our laboratory (Supplementary Fig. S1)^21,22^. *leg1a*^*zju1/zju1*^ single (*leg1a_mu*), *leg1b*^*zju1/zju1*^ single (*leg1b_mu*) and *leg1a*^*zju3/zju3*^*leg1b*^*zju1/zju1*^ double (*leg1_dm*) homozygous mutants were all viable and fertile. Whole-mount in situ hybridization (WISH) using *fabp10a* as a molecular marker for the liver at 3.5 dpf showed that, compared with the wild-type controls (WT), only the *leg1a*^*zju3/zju3*^*leg1b*^*zju1/zju1*^ double mutant exhibited a smaller liver with neither *leg1a*^*zju1/zju1*^ or *leg1b*^*zju1/zju1*^ single mutants displaying such a phenotype (Fig. 1a, b). This seemed to confirm the redundant function of *leg1a* and *leg1b* in embryonic liver development^20^. Double-probe WISH (using either *fabp10a* and *trypsin* probes together, or *fabp10a* and *fabp2* probes together at 3.5 dpf) revealed that both the exocrine pancreas (as marked by *trypsin*) (Fig. 1c, d) and the intestine (as marked by *fabp2*) (Fig. 1e, f) exhibited a significant size reduction corresponding to that of the liver, but again this occurred only in the *leg1a*^*zju3/zju3*^*leg1b*^*zju1/zju1*^ double mutants. Interestingly, when comparing either the liver and exocrine pancreas (Fig. 1d, for ‘exocrine pancreas to liver ratio’) or the liver and intestine (Fig. 1f, for ‘intestine to liver ratio’) the scale of size reduction in the *leg1a*^*zju3/zju3*^*leg1b*^*zju1/zju1*^ double mutants was similar for both comparison, consistent with the assumption that Leg1a and Leg1b may act as a secreted signaling molecule^20^ active in regulating the development of multiple organs/tissues^21^.

### The HDGCR is activated in both *leg1a* and *leg1b* single mutants

**Fig. 2:**
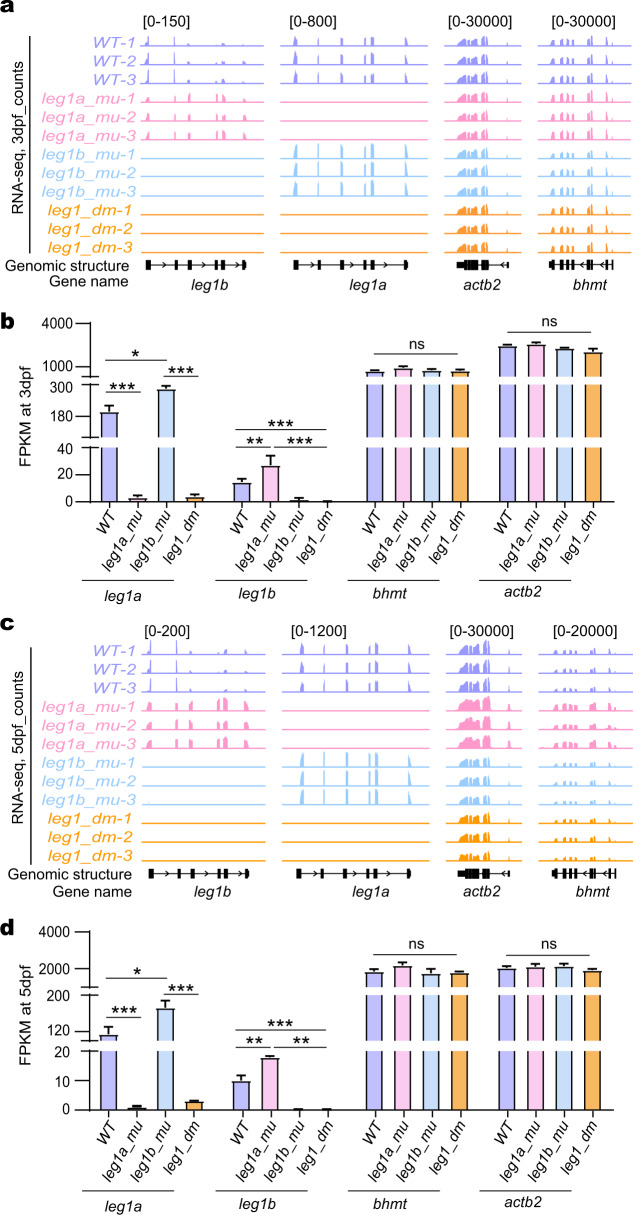
RNA-seq analysis revealed activation of GCR in both *leg1a*^*zju1/zju1*^ single and *leg1b*^*zju1/zju1*^ single mutants. **a**–**d** Genome browser view (**a**, **c**) and statistical analysis (**b**, **d**) of *leg1a* and *leg1b* transcript counts (FPKM) together with *actb2* and *bhmt* two control genes in RNA-seq samples obtained from WT, *leg1a_mu* single, *leg1b_mu* single and *leg1_dm* double mutant embryos at 3 dpf (**a**, **b**) and at 5 dpf (**c**, **d**), respectively. ns, no significance; *P < 0.05; **P < 0.01; ***P < 0.001.

Next, we performed an RNA-seq experiment using the total RNA extracted from WT, *leg1a*^*zju1/zju1*^ single (*leg1a_mu*), *leg1b*^*zju1/zju1*^ single (*leg1b_mu*), and *leg1a*^*zju3/zju3*^*leg1b*^*zju1/zju1*^ double (*leg1_dm*) homozygous mutants at both 3 dpf and 5 dpf. The obtained RNA-seq data were of satisfactory quality based on the analysis of the volume of clean bases, the Clean Q30 Bases Rate, the mapping rate of the clean sequences to the zebrafish genome (Danio rerio.GRCz11) (Supplementary Tables S1, S2), and principal component analysis (PCA) (Supplementary Fig. S2a, b). We first compared the transcript levels of *leg1a* and *leg1b* and also two control genes, *actin beta 2* (*actb2*) and *betaine homocysteine S-methyltransferase* (*bhmt*), in these four genotypes. Based on analyzing fragments per kilobase of exon model per million mapped fragments (FPKM), we found that the *leg1a* transcript levels were extremely low in *leg1a*^*zju1/zju1*^ single and *leg1a*^*zju3/zju3*^*leg1b*^*zju1/zju1*^ double mutant, so did *leg1b* in *leg1b*^*zju1/zju1*^ single and *leg1a*^*zju3/zju3*^*leg1b*^*zju1zju1*^ double mutants, at both 3 dpf (Fig. 2a, b) and 5 dpf (Fig. 2c, d). The results suggested that *leg1a*^*zju1*^, *leg1a*^*zju3*^, and *leg1b*^*zju1*^ mutant mRNAs (all bearing a PTC) were subjected to degradation by the NMD pathway^14^. On the other hand, compared with WT controls, the levels of *leg1a* transcripts were approximately 50% and 44% higher in the *leg1b*^*zju1/zju1*^ mutant and the levels of *leg1b* transcripts were 1.8- and 1.9-old higher in the *leg1a*^*zju1/zju1*^ mutants at 3 dpf (Fig. 2b) and 5 dpf (Fig. 2d), respectively. No significant changes were observed for *actb2* and *bhmt* in any of these samples. These results suggest that the HDGCR is likely activated in both *leg1a*^*zju1/zju1*^ and *leg1b*^*zju1/zju1*^ single mutants.

**Fig. 3:**
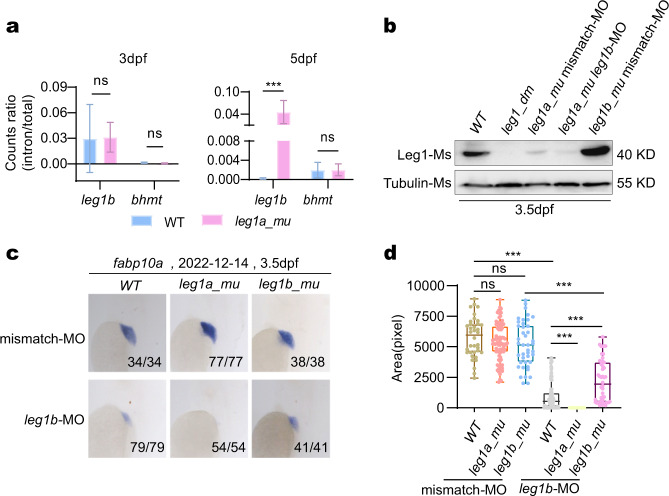
Knockdown of *leg1b* by MO in *leg1a*^*zju1/zju1*^ caused a small liver phenotype. **a** Statistical analysis of the ratios of the precursor and total *leg1b* transcripts in WT and *leg1a*^*zju1/zju1*^ at 3 dpf and 5 dpf, respectively. Nine RNA-seq datasets each for WT and *leg1a*^*zju1/zju1*^ at 3 dpf and 5 dpf were used for this analysis (refer to Supplementary Tables S1, S2, S7, S8, S13, S14). **b** Western blot of Leg1 from embryos at 3.5 dpf after injection of *leg1b*-MO in different genotypes as shown. Tubulin, loading control. **c**, **d** Representative images showing the WISH result using the *fabp10a* probe (**c**) and statistical analysis of the liver size (**d**) in different genotypes injected with *leg1b*-MO or control mismatch morpholino (mismatch-MO) as indicated. **c** Top: the date of experiments performed; bottom right: number of embryos showing the phenotype over total embryos examined. **d** The *y* axis shows the positive signal area in each embryo stained with the *fabp10a* probe.

To determine whether the HDGCR was indeed activated in *leg1a*^*zju1/zju1*^, we compared the ratios of the *leg1b* precursor RNA (defined by containing intron sequences) vs total *leg1b* transcripts in the 36 independent RNA-seq datasets, including 9 datasets each for WT and *leg1a*^*zju1/zju1*^ at both 3 dpf and 5 dpf (Supplementary Tables S1, S2, S7, S8, S13, S14), with *bhmt* as the control. The scenario was that the ratio of precursor RNA (un-spliced or partially spliced) could serve as an index for transcription activity^25^. The result showed that the ratio of the *leg1b* precursor RNA vs total *leg1b* transcripts in *leg1a*^*zju1/zju1*^ was significantly higher than that in the WT at 5 dpf, reaching ~267 fold that of the WT and where no significant difference was observed for the *bhmt* gene (Fig. 3a, right panel). This suggested activation of *leg1b* transcription in *leg1a*^*zju1/zju1*^ mutants. Interestingly, no significant difference was observed at 3 dpf (Fig. 3a, left panel).

Next, we knocked down the Leg1b expression by injecting the morpholino (*leg1b*-MO) which specifically targeted the start codon ATG region of the *leg1b* mRNA^20^ into the WT, *leg1a*^*zju1/zju1*^ and *leg1b*^*zju1/zju1*^ embryos at the one-cell stage, and then harvested the embryos at 3.5 dpf for protein extraction and WISH assay. The hypothesis here was that knockdown of the upregulated *leg1b* in *leg1a*^*zju1/zju1*^ would lead to a small liver phenotype. Western blot assay failed to detect total Leg1 proteins in the *leg1a*^*zju3/zju3*^*leg1b*^*zju1zju1*^ double mutant embryos (Fig. 3b). This suggested *leg1a*^*zju3/zju3*^ and *leg1b*^*zju1/zju1*^ both to be null alleles. Thus, it was logical to put forward that the total Leg1 proteins detected in *leg1a*^*zju1/zju1*^ represented Leg1b protein only, and vise versa, Leg1a protein only in *leg1b*^*zju1/zju1*^. The *leg1a*^*zju1/zju1*^ embryos contained a lower level of total Leg1 compared with WT. This is likely an outcome of the depletion of Leg1a which is the predominant form at the embryonic stage (*leg1a* vs *leg1b* FPKM, 114.9 vs 10 at 3 dpf, and 198.3 vs 14.6 at 5 dpf). The Leg1b level was further lowered by *leg1b*-MO injection in the *leg1a*^*zju1/zju1*^ embryos (Fig. 3b). Notably, the Leg1a protein level in *leg1b*^*zju1/zju1*^ was upregulated when compared with WT (Fig. 3b). Consistent with our previous report^20^, the WISH result showed that the *leg1b*-MO injection had resulted in a smaller liver than that of the WT embryos. Strikingly, the *leg1b*-MO injection into the *leg1a*^*zju1/zju1*^ embryos caused an almost liverless phenotype, which was far more severe than the *leg1b*-MO injected WT (Fig. 3c, d). Interestingly, injecting the *leg1b*-MO into the *leg1b*^*zju1/zju1*^ mutant also caused a small liver phenotype. However, this remained significantly bigger than that in the WT embryos injected with *leg1b*-MO (Fig. 3c, d), possibly due to the binding of the *leg1b*-MO to the *leg1b*^*zju1/zju1*^ mutant mRNA which may partially compromise the upregulation of *leg1a* through the HDGCR.

### *upf1* and *upf3a* transcript levels were not significantly altered in either *leg1a* or *leg1b* single mutants

**Fig. 4:**
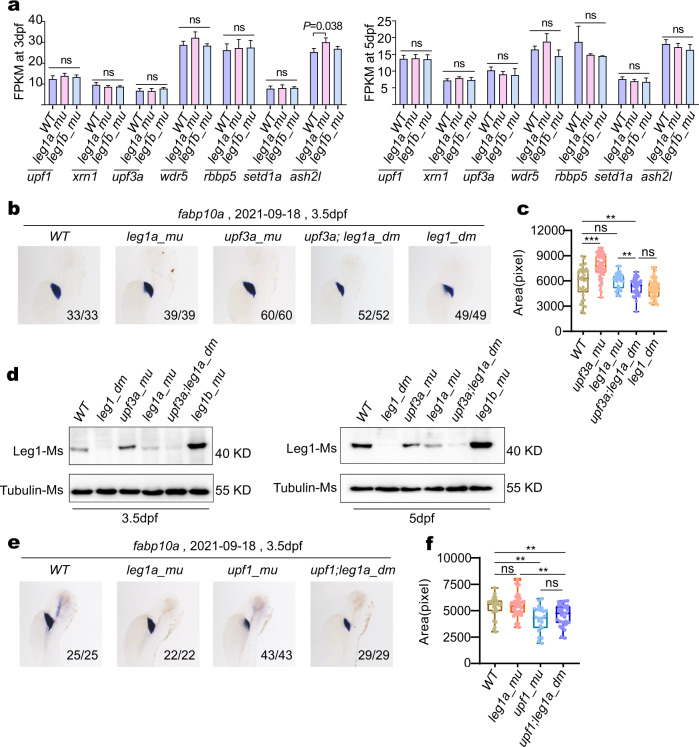
*upf3a*^*−/−*^*leg1a*^*zju1/zju1*^ double mutant exhibits a smaller liver phenotype. **a** Statistical analysis of transcript counts (FPKM) of the genes encoding components of the NMD pathway and COMPAS complex in RNA-seq samples obtained from WT, *leg1a_mu* single and *leg1b_mu* single mutant embryos at 3 dpf and at 5 dpf, respectively. **b**, **c** Representative images showing the WISH result using the *fabp10a* probe (**b**) and the statistical analysis of liver sizes (**c**) in the WT, *leg1a_mu*, *upf3a_mu*, *upf3a;leg1a_dm* and *leg1_dm* embryos at 3.5 dpf. **d** Western blot of Leg1 protein in different genotypes at 3.5 dpf and 5 dpf, respectively. Tubulin, loading control. **e**, **f** Representative images showing the WISH result using the *fabp10a* probe (**e**) and the statistical analysis of liver sizes (**f**) in the WT, *leg1a_mu*, *upf1_mu,* and *upf1;leg1a_dm* embryos at 3.5dpf. **b**, **e** Top: the date of experiments performed; bottom right: number of embryos showing the phenotype over total embryos examined. ns, no significance; **P < 0.01; ***P < 0.001.

To explore whether any common molecular signaling or biological pathway(s) might be mobilized to trigger the HDGCR in *leg1a*^*zju1/zju1*^ and *leg1b*^*zju1/zju1*^ single mutants, we carried out a further analysis of the RNA-seq data. Firstly, we checked the transcript levels of genes that had previously been shown to be involved in mediating the HDGCR (*upf1*, *xrn1*, *upf3a*, *wdr5*, *rbbp5*, *setd1a,* and *ash2l*)^12,13^, in the RNA-seq data. No significant difference was found for any of these genes compared to WT, except *ash2l* (*P* = 0.038) in *leg1a*^*zju1/zju1*^ at 3 dpf (Fig. 4a), suggesting that the transcriptional regulation of these genes might not be a prerequisite for the HDGCR activation in *leg1a*^*zju1/zju1*^ and *leg1b*^*zju1/zju1*^ single mutants.

Next, we analyzed differentially expressed genes (DEGs) by focusing on the RNA-seq data obtained from the larvae at 3 dpf (as the HDGCR had been observed at this time point) (Fig. 2). 1284 downregulated and 570 upregulated DEGs in *leg1a*^*zju1/zju1*^ and 696 downregulated and 659 upregulated DEGs in *leg1b*^*zju1/zju1*^ were identified (Supplementary Tables S3, S4). *leg1a*^*zju1/zju1*^ and *leg1b*^*zju1/zju1*^ shared 334 downregulated and 182 upregulated DEGs (Supplementary Fig. S2c). We then undertook gene ontology (GO) analysis of the 334 shared downregulated DEGs. Those significantly affected processes under the terms of molecular function (MF) and biologic process (BP) were mainly related to metabolic activities, while genes with a product related to the extracellular space and region were found under the cellular component term (CC) (Supplementary Fig. S2d). Analysis of the 182 shared upregulated DEGs identified a few metabolic pathways under the MF and BP terms, however, with only a handful of genes (Supplementary Fig. S2e). To gain more insight into the 182 shared upregulated DEGs, we manually searched the known or putative functions of these DEGs in the Zebrafish Information Network (ZFIN) (https://zfin.org/). We noticed that 23 of these DEGs were related to DNA-binding and transcription regulation (Supplementary Fig. S2f). However, whether the upregulation of these 23 DEGs was a reason for, or a consequence of, the HDGCR, or due to the loss-of-function of Leg1a/Leg1b remains unknown. This is primarily due to a number of factors: (1) the expression of *leg1a* and *leg1b* is relatively liver-specific, so the HDGCR induced by *leg1a*^*zju1/zju1*^ or *leg1b*^*zju1/zju1*^ mutant mRNA is presumably restricted to within the liver cells; (2) while the RNA samples were prepared from the whole larvae at 3 dpf, when the liver volume vs that of the whole larvae is less than 0.1%^26^, detection of the HDGCR-related gene transcripts may be obscured; and (3) Leg1a and Leg1b are secretory proteins whose function might not be limited to the liver, and thus the loss-of-function of *leg1a* or *leg1b* might affect gene expression profiles in multiple organs/tissues.

To gain an insight into the biological function of Leg1 in zebrafish, we then compared the RNA-seq data from the WT and *leg1a*^*zju3/zju3*^*leg1b*^*zju1/zju1*^ double mutant samples. DEseq2 analysis identified 904 DEGs (378 upregulated and 526 downregulated) at 3 dpf and 875 DEGs (453 upregulated and 422 downregulated) at 5dpf (*leg1_dm* vs WT:|log_2_(fold-change) |≥ 1, *p*_*ad j*_ < 0.05) (Supplementary Tables S3–S6). GO analysis revealed the ‘intracellular signal transduction’ category under the ‘BP’ term to be the process most significantly affected in the *leg1a*^*zju3/zju3*^*leg1b*^*zju1/zju1*^ double mutant for both downregulated and upregulated DEGs at both 3 dpf and 5 dpf (Supplementary Fig. S3a, b). The ‘intracellular signal transduction’ process contained 27 genes at 3 dpf and 34 genes at 5d pf of the downregulated DEGs (3 dpf and 5 dpf sharing 21 genes) (Supplementary Fig. S3c) and 24 genes at 3 dpf and 26 genes at 5 dpf of the upregulated DEGs (3 dpf and 5 dpf sharing 15 genes) (Supplementary Fig. S3d). Surprisingly, many of these genes had not been genuinely annotated and were only assigned with ENSEMBL gene identity numbers (https://asia.ensembl.org/index.html). Based on cross-species homology analysis, we noticed that a large proportion of the above genes, regardless of whether downregulated or upregulated, were predicted to encode proteins with putative ATP-binding activity and show homology to the NOD-like receptor pyrin domain-containing (Nlrp) family proteins (Supplementary Fig. S3c, d). Nlrp proteins play important roles in mediating the innate immune response upon pathogen infections^27–29^. GO analysis, therefore, suggested that Leg1 might act as a secreted signaling molecule active in regulating the innate immune response and other biological processes.

### *upf3a* and *leg1a* double mutations cause a small liver phenotype

**Fig. 5:**
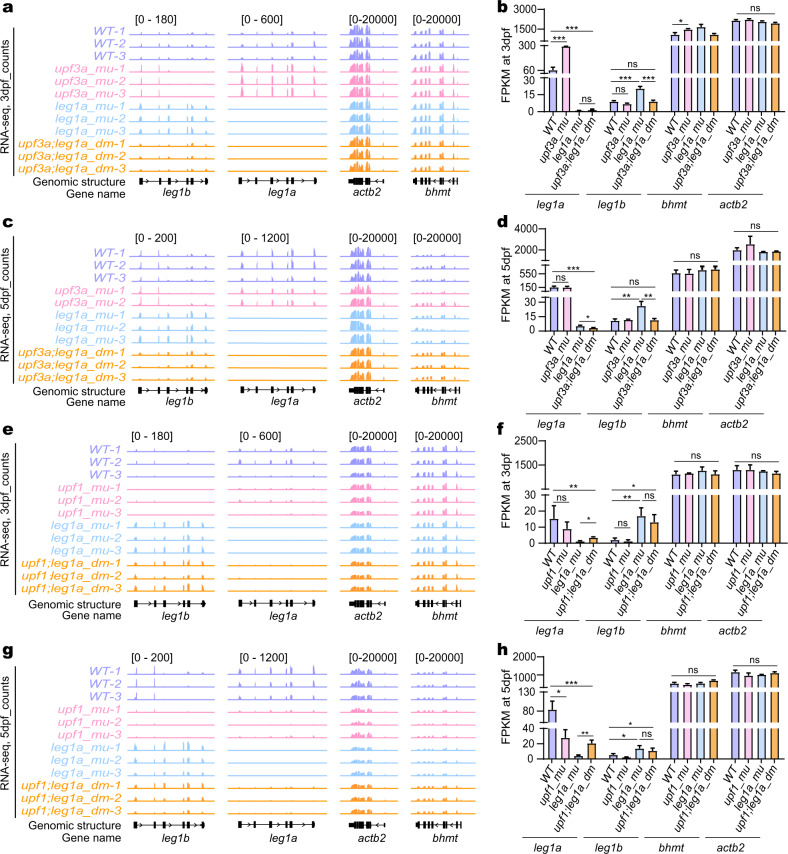
RNA-seq analysis showed that the upregulated *leg1b* transcript level in *leg1a*^*zju1/zju1*^ was downregulated by Upf3a depletion but was unaffected by Upf1 depletion. **a**–**d** Showing genome browser view (**a**, **c**) and statistical analysis (**b**, **d**) of *leg1a* and *leg1b* transcript counts (FPKM) together with *actb2* and *bhmt* two control genes in RNA-seq samples obtained from WT, *upf3a*^*−/−*^ (*upf3a_mu*) single, *leg1a*^*zju1/zju1*^ (*leg1a_mu*) single and *upf3a*^*−/−*^*leg1a*^*zju1/zju1*^ (*upf3a;leg1a_dm*) double mutant embryos at 3 dpf (**a**, **b**) and at 5 dpf (**c**, **d**), respectively. **e**–**h** Genome browser view (**e**, **g**) and statistical analysis (**f**, **h**) of *leg1a* and *leg1b* transcript counts (FPKM) together with *actb2* and *bhmt* two control genes in RNA-seq samples obtained from WT, *upf1*^*−/−*^ (*upf1_mu*) single, *leg1a*^*zju1/zju1*^ (*leg1a_mu*) single and *upf1*^*−/−*^*leg1a*^*zju1/zju1*^ (*upf1;leg1a_dm*) double mutant embryos at 3 dpf (**e**, **f**) and at 5 dpf (**g**, **h**), respectively. ns, no significance; *P < 0.05; **P < 0.01; ***P < 0.001.

To determine the role of *upf3a* and *upf1* in the HDGCR in *leg1a*^*zju1/zju1*^, we obtained *upf3a*^*−/−*^*leg1a*^*zju1/zju1*^ (*upf3a;leg1a_dm*) and *upf1*^*−/−*^*leg1a*^*zju1/zju1*^ (*upf1;leg1a_dm*) double mutants, respectively (Supplementary Fig. S4a, b). The *upf3a*^*−/−*^*leg1a*^*zju1/zju1*^ double mutant was viable and fertile, just as were the *upf3a*^*−/−*^ single (*upf3a_mu*) and *leg1a*^*zju1/zju1*^ single (*leg1a_mu*) mutants^13,21^. However, the *upf1*^*−/−*^*leg1a*^*zju1/zju1*^ double mutant showed embryonic lethality, just as did the *upf1*^*−/−*^ single (*upf1_mu*) mutant^13^. Consistent with our previous report^13^, *upf3a*^*−/−*^ mutants displayed a slightly larger liver than that in the WT at 3.5 dpf as revealed by WISH using an *fabp10a* probe (Fig. 4b, c). Interestingly, the maternal-zygotic *upf3a*^*−/−*^*leg1a*^*zju1/zju1*^ embryos developed small livers, resembling the *leg1a*^*zju3/zju3*^*leg1b*^*zju1zju1*^ double mutant (Fig. 4b, c). Considering the fact that the *leg1a*^*zju1/zju1*^ mutant developed a normal-sized liver and the *upf3a*^*−/−*^ mutant with a slightly enlarged liver, the small liver phenotype displayed by the *upf3a*^*−/−*^*leg1a*^*zju1/zju1*^ double mutant is proposed to be the outcome of a compromised HDGCR leading to a reduction in *leg1b* expression in the *upf3a*^*−/−*^*leg1a*^*zju1/zju1*^ double mutant. This hypothesis was supported by the RNA-seq data analysis (Fig. 5a–d) and by our western blot assay which showed that, compared with the *leg1a*^*zju1/zju1*^ single mutant, the Leg1b protein level was reduced in the *upf3a*^*−/−*^*leg1a*^*zju1/zju1*^ double mutant (Fig. 4d). However, we cannot currently exclude the possibility that a concomitant loss-of-function of *upf3a* and *leg1a* might had an accumulative effect to cause the observed small liver phenotype. The *upf1*^*−/−*^*leg1a*^*zju1/zju1*^ double mutant, resembling the *upf1*^*−/−*^ single mutant^13^, exhibited smaller livers compared to WT(Fig. 4e, f). This suggests that the *upf1* deleterious mutation is genetically epistatic to *leg1a*^*zju1/zju1*^, despite the continued upregulation of *leg1b* expression in the *upf1*^*−/−*^*leg1a*^*zju1/zju1*^ double mutant (Fig. 5e–h).

### Depletion of Upf3a but not Upf1 abolishes the upregulation of *leg1b* caused by the *leg1a* mutation

Total RNA extracted from WT, *leg1a*^*zju1/zju1*^ single (*leg1a_mu*), *upf3a*^*−/−*^ single (*upf3a_mu*) and *upf3a*^*−/−*^*leg1a*^*zju1/zju1*^ double mutant (*upf3a;leg1_dm*) at 3 dpf and 5 dpf (three independent samples for each genotype) was subjected to RNA-seq analysis (Supplementary Fig. S5a, b and Tables S7–S12). PCA analysis revealed that, except for one *upf3a*^*−/−*^ sample at 5 dpf, three independent samples for each genotype were neatly clustered (Supplementary Fig. S5a, b). The one non-clustered sample was excluded from further analysis. Expression analysis based on FPKM revealed that the *leg1a* mutant mRNA was at a very low level, not only in the six *leg1a*^*zju1/zju1*^ single mutant samples, but also in the six *upf3a*^*−/−*^*leg1a*^*zju1/zju1*^ double mutant samples when compared to the WT samples at 3 dpf and 5 dpf. No significant changes were found relating to the two control genes *bhmt* and *actb2* (Fig. 5a–d). This suggested that the *leg1a* mutant mRNA had been subjected to degradation by an active NMD pathway in these genetic backgrounds, and thus confirmed Upf3a not to be a positive regulator of the NMD pathway^17^. In contrast, the *leg1b* mRNA levels were upregulated in all six *leg1a*^*zju1/zju1*^ single mutant samples as compared to the WT samples at both 3 dpf and 5 dpf (Fig. 5a–d). However, the upregulated *leg1b* expression was significantly downregulated in the six *upf3a*^*−/−*^*leg1a*^*zju1/zju1*^ double mutant samples as compared to the *leg1a*^*zju1/zju1*^ single mutant and was returned to a similar level of that in the WT at both 3 dpf and 5 dpf (Fig. 5a–d). This suggested that Upf3a depletion had compromised the HDGCR in *leg1a*^*zju1/zju1*^.

Three independent RNA samples from WT, *leg1a*^*zju1/zju1*^ single (*leg1a_mu*), *upf1*^*−/−*^ single (*upf1_mu*) and *upf1*^*−/−*^*leg1a*^*zju1/zju1*^double (*upf1;leg1a_dm*) mutant at 3 dpf and 5 dpf were also subjected to RNA-seq analysis (Supplementary Fig. S5c, d and Tables S13–S18). We again observed a very low level of *leg1a* mutant mRNA in the six *leg1a*^*zju1/zju1*^ single mutant samples at 3 dpf and 5 dpf (Fig. 5e–h). In contrast, the levels of *leg1a* mutant mRNA were significantly higher in the *upf1*^*−/−*^*leg1a*^*zju1/zju1*^ double mutant than in the *leg1a*^*zju1/zju1*^ single mutant (Fig. 5e–h), likely due to the inactivation of the NMD pathway after depleting Upf1 in the *leg1a*^*zju1/zju1*^ background^14^. Expression analysis also revealed the upregulation of the *leg1b* expression in all six *leg1a*^*zju1/zju1*^ single mutant samples compared to the WT samples at 3 dpf and 5 dpf (Fig. 5e–h). Notably, the *leg1b* mRNA levels in the *upf1*^*−/−*^*leg1a*^*zju1/zju1*^ double mutant were not significantly different from those in the *leg1a*^*zju1/zju1*^ single mutant (Fig. 5e–h), suggesting that Upf1 does not play an obvious role in the activation of the HDGCR in *leg1a*^*zju1/zju1*^.

**Fig. 6:**
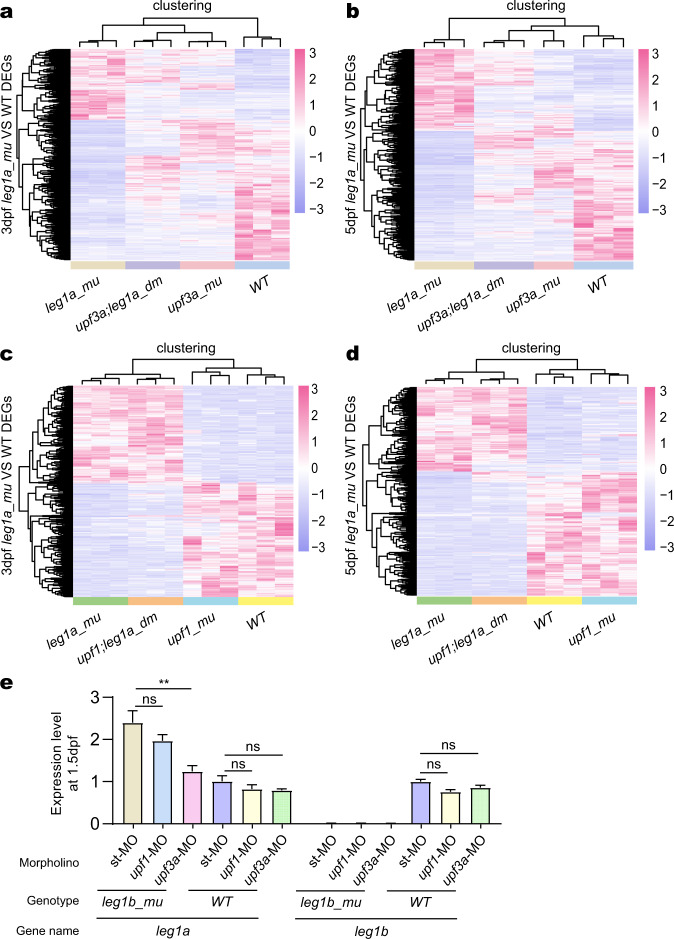
Depletion of Upf3a but not of Upf1 obviously alters the DEGs in the *leg1a*^*zju1/zju1*^ mutant. **a**, **b** DEGs between WT and *leg1a*^*zju1/zju1*^ (*leg1a_mu*) at 3 dpf (**a**) and 5 dpf (**b**) were identified by analyzing the RNA-seq data, respectively. The heat-map shows the hierarchical clustering of these DEGs in the RNA-seq samples obtained from *upf3a*^*−/−*^ (*upf3a_mu*) single and *upf3a*^*−/−*^*leg1a*^*zju1/zju1*^ (*upf3a;leg1a_dm*) double mutant embryos at 3 dpf (**a**) and 5 dpf (**b**), respectively. **c**, **d** DEGs between WT and *leg1a*^*zju1/zju1*^ (*leg1a_mu*) at 3 dpf (**a**) and 5dpf (**b**) were identified by analyzing the RNA-seq data, respectively. The heat-map shows the hierarchical clustering of these DEGs in the RNA-seq samples obtained from *upf1*^*−/−*^ (*upf1_mu*) single and *upf1*^*−/−*^*leg1a*^*zju1/zju1*^ (*upf1;leg1a_dm*) double mutant embryos at 3 dpf (**c**) and 5 dpf (**d**), respectively. **e** The qPCR analysis of *leg1a* and *leg1b* transcripts in WT and *leg1b*^*zju1/zju1*^ at 1.5 dpf. The embryos were injected with st-MO, *upf1*-MO, or *upf3a*-MO at the one-cell stage.

Strikingly, further hierarchical analysis of the 366 upregulated and 718 downregulated DEGs at 3 dpf, and 473 upregulated and 746 downregulated DEGs at 5 dpf between the *leg1a*^*zju1/zju1*^ mutant and WT identified by the RNA-seq experiment (Supplementary Tables S9–S12), showed that 263 (3 dpf) and 327 (5 dpf) upregulated and 493 (3 dpf) and 520 (5 dpf) downregulated DEGs were obviously deregulated in the *upf3a*^*−/−*^*leg1a*^*zju1/zju1*^ double mutant. In contrast, the expression patterns of the majority of the DEGs identified in the *leg1a*^*zju1/zju1*^ single mutant were not drastically altered in the *upf1*^*−/−*^*leg1a*^*zju1/zju1*^ double mutant (Fig. 6a–d). These data suggest that depleting Upf3a in *leg1a*^*zju1/zju1*^ had blocked the HDGCR and altered gene expression profiles. Interestingly, GO analysis of the DEGs altered by the Upf3a depletion at 3 dpf (Supplementary Tables S9, S10) showed that some metabolic pathways under the GO_MF and GO_BP terms and ‘membrane’ and ‘integral component of membrane’ categories under the GO_CC term were significantly affected for the altered downregulated DEGs (Supplementary Fig. S5e). However, only the ‘extracellular space’ category under the GO_CC term was outstanding for the altered upregulated DEGs (Supplementary Fig. S5f). At 5 dpf, ‘RNA polymerase II transcription factor activity’ and ‘RNA polymerase II regulatory region’ were two categories identified for both the downregulated and upregulated DEGs altered by Upf3a depletion (Supplementary Fig. S5g, h) but each constituting of distinct genes (Supplementary Tables S11, S12). Meanwhile, the ‘intracellular signal transduction’ category for the altered downregulated DEGs and the ‘extracellular space/region’ and ‘mitochondrion’ categories for the altered upregulated DEGs at 5 dpf (Supplementary Tables S11, S12) were also identified by the GO analysis (Supplementary Fig. S5g, h). However, how and whether these DEGs were affected by the Upf3a depletion remains unknown.

It is intriguing that *upf3a*^*−/−*^ and *upf1*^*−/−*^ had distinct effect upon DEGs in *leg1a*^*zju1/zju1*^ where both *upf3a*^*−/−*^*leg1a*^*zju1/zju1*^ and *upf1*^*−/−*^*leg1a*^*zju1/zju1*^ double mutants displayed a small liver phenotype (Fig. 4b, c, e, f). This implies that distinct liver developmental genes might be altered by *upf3a*^*−/−*^ and *upf1*^*−/−*^. Zebrafish liver development is governed by a genetic network formed by genes encoding multiple signaling molecules (e.g., Bmp, Fgf and Wnt, etc.) and transcription factors (such as Foxa and Gata family members)^30–33^. When compared with the WT, analysis of the RNA-seq data identified 647 upregulated and 933 downregulated DEGs in *upf3a*^*−/−*^*leg1a*^*zju1/zju1*^ double mutant, and 1264 upregulated and 766 downregulated in *upf1*^*−/−*^*leg1a*^*zju1/zju1*^ double mutant at 3 dpf (Supplementary Tables S9, S10, S15, S16). Cross-comparison showed that *upf3a*^*−/−*^*leg1a*^*zju1/zju1*^ and *upf1*^*−/−*^*leg1a*^*zju1/zju1*^ double mutants shared 74 upregulated and 121 downregulated DEGs, respectively. Despite this, no well-known genes controlling liver development were among these shared genes (Supplementary Fig. S6a). We then examined the distinct downregulated DEGs and found that genes related to the BMP signaling (*bmp2a*, *bmp3,* and *bmp7b*) and Fgf signaling (*fgf12a*) were significantly downregulated in *upf1*^*−/−*^*leg1a*^*zju1/zju1*^ double mutant whereas signaling molecules *fgf1b* and *fgf20b* and transcription factors *hnf1a*, *gata1a,* and *pparaa* were identified in *upf3a*^*−/−*^*leg1a*^*zju1/zju1*^ double mutant (Supplementary Fig. S6b, c).

To discover whether Upf3a is also required for the compensatory expression of *leg1a* in the *leg1b*^*zju1/zju1*^ mutant, we injected *upf1* and *upf3a* specific MOs^13^ (Supplementary Fig. S7) into the fertilized WT and *leg1b*^*zju1/zju1*^ eggs at the one-cell stage and harvested the embryos at 1.5 dpf for total RNA extraction. Quantitative rea-time PCR (qPCR) analysis of the *leg1a* transcripts showed that *upf3a*-specific MO but not the *upf1*-specific MO significantly downregulated the *leg1a* expression in the *leg1b*^*zju1/zju1*^ mutant (Fig. 6e).

### Upregulation of *leg1b* caused by the *leg1a* mutation is not coupled with H3K4me3 modification

**Fig. 7:**
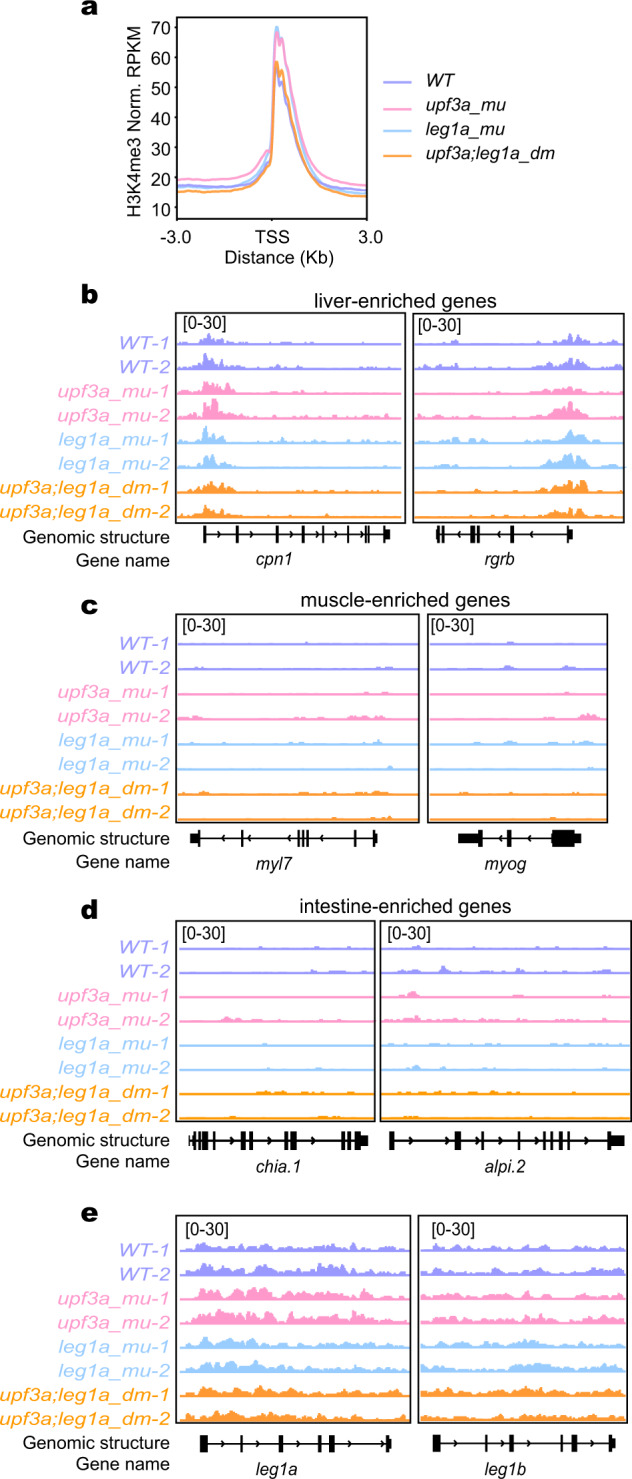
Upregulation of *leg1b* in *leg1a*^*zju1/zju1*^ is not coupled with an enrichment of H3K4me3 around its TSS. **a** Graph showing the normalized reads distribution profiles of the genome-wide H3K4me3 enrichment (RPKM) in the liver dissected from WT, *leg1a_mu*, *upf3a_mu* and *upf3a;leg1a_dm* embryos at 5 dpf. TSS, transcription start site. **b**–**e** Genome browser view of the H3K4me3 enrichment in the genomic region of representative liver-specific (**b**), muscle-enriched (**c**), intestine-enriched genes (**d**) and *leg1a* and *leg1b* (**e**) genes. The *y* axis shows the ULI-NChIP counts across the genomic region shown.

Upf3a is proposed to interact with Wdr5 (a COMPASS component) to mediate the HDGCR through enhancing H3K4me3 in the promoter region of compensatory genes^13^. We, therefore, micro-dissected the liver buds from WT, *leg1a*^*zju1/zju1*^ single (*leg1a_mu*), *upf3a*^*−/−*^ single (*upf3a_mu*), and *upf3a*^*−/−*^*leg1a*^*zju1/zju1*^ double (*upf3a;leg1a_dm*) mutant embryos at 5 dpf (Supplementary Fig. S8a) for ULI-NChIP-seq analysis of the distribution of H3K4me3 on chromatin (Supplementary Table S19). Normalized reads distribution profiles showed that H3K4me3 was enriched around the transcription start sites (TSS) of 11,197 genes (Fig. 7a; Supplementary Table S20), including housekeeping genes *actb2*, *pck1* and *gapdh* and liver-enriched *cpn1*, *rgrb*, *gstp1* and *tpt1* in all four genotypes (Fig. 7b; Supplementary Fig. S8b, c). As expected, H3K4me3 was not enriched around the TSS sites of the muscle gene *myl7*, *myog*, *myod1* and *myoz2b* or the intestine gene *chia.1* and *alpi.2* in the liver cells (Fig. 7c, d; Supplementary Fig. S8d). Surprisingly, ULI-NChIP-seq results did not reveal any obvious changes in the level of H3K4me3 around the TSS region of *leg1b* among all four genotypes. There was also no specific TSS-enrichment of H3K4me3 for either the *leg1a* or *leg1b* gene (Fig. 7e).

**Fig. 8:**
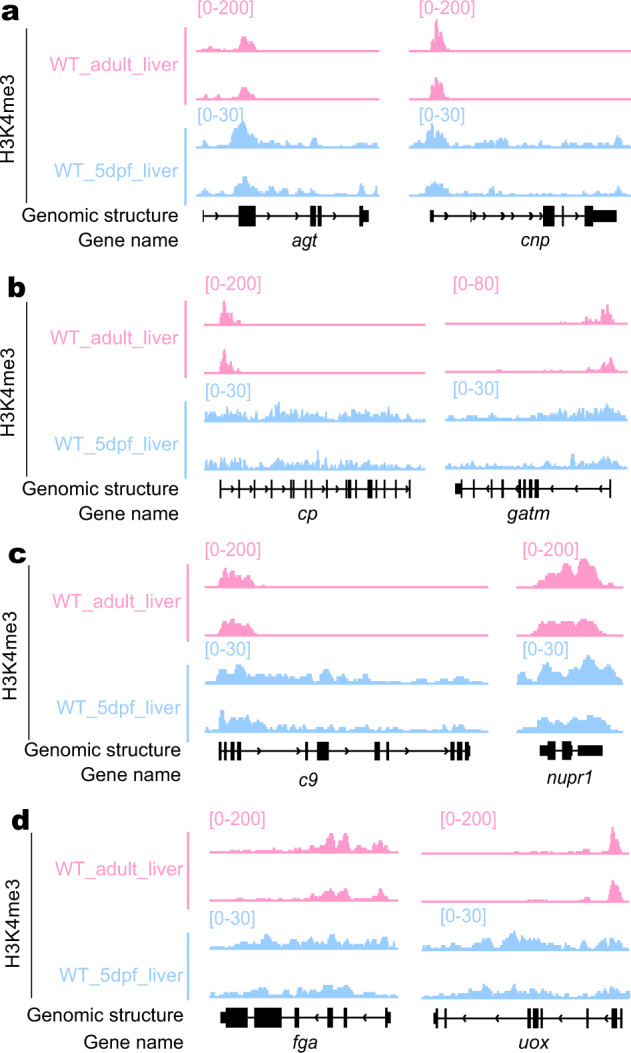
Cross-comparison of H3K4me3 enrichment in the genomic regions of 70 liver-enriched genes between WT adult and embryonic liver. **a**–**d** Genome browser view of the genomic region of representative genes in the category of TSS specific-enrichment of H3K4me3 in both adult and embryonic liver (**a**), TSS-specific enrichment of H3K4me3 in the adult liver but not the embryonic liver (**b**), H3K4me3 enrichment in the TTS together with gene body region in both adult and embryonic liver (**c**) and different patterns of H3K4me3 enrichment in the TSS together with gene body region between adult and embryonic liver (**d**). The *y* axis shows the ChIP-seq counts across the genomic region shown. The adult liver ChIP-seq data were extracted from the database (SRA: SRX6422954 and SRX6422955) and the embryonic liver data were obtained in this work.

To investigate whether H3K4me3 modification around the TSS region is a hallmark for the expression of liver-enriched genes, we compared the patterns of H3K4me3 modification between the adult and embryonic liver on the genomic regions of the 70 genes (the expression of which had been experimentally proved to be enriched in both adult and embryonic liver and are therefore termed as liver-enriched genes, including *leg1a* and *leg1b*) (Supplementary Table S21)^19^. The zebrafish adult liver ChIP-seq data was extracted from the available public database^34^. The result showed that among the 47 liver-enriched genes showing an obvious TSS-enrichment of H3K4me3 in the adult liver only 27 (including *agt* and *cnp*) displayed enrichment (Fig. 8a; Supplementary Fig. S9a), while another 20 (including *leg1a*, *leg1b*, *cp,* and *gatm*) lacked any obvious TSS-enrichment in the embryonic liver (Figs. 7e and 8b; Supplementary Fig. S9b). For the 22 liver-enriched genes displaying an H3K4me3 enrichment across the TSS and along the adjacent gene body region in the adult liver, 13 genes (including *c9* and *nupr1*) showed similar H3K4me3 enrichment patterns (Fig. 8c; Supplementary Fig. S9c) while the remaining 9 genes (inclduing *fga* and *uox*) were drastically different in the embryonic liver (Fig. 8d; Supplementary Fig. S9d). For one gene, *zar1*, no H3K4me3 enrichment was detected in the adult liver, whilst such enrichment was observed in the embryonic liver (Supplementary Fig. S9e).

## Discussion

In this report, we have shown that *leg1b* is robustly upregulated in the *leg1a*^*zju1/zju1*^ mutant. This clearly suggested that the activation of the HDGCR had been triggered by the *leg1a* deleterious mutation. RNA-seq analysis of the *upf3a*^*−/−*^*leg1a*^*zju1/zju1*^ and *upf1*^*−/−*^*leg1a*^*zju1/zju1*^ double mutants implicated that Upf3a, but not Upf1, was necessary for the HDGCR triggered by the *leg1a*^*zju1/zju1*^ deleterious mutation. This role of Upf3a was supported by the small liver phenotype displayed by the *upf3a*^*−/−*^*leg1a*^*zju1/zju1*^ double mutant, mimicking the liver phenotype observed in the *leg1a*^*zju3/zju3*^*leg1b*^*zju1/zju1*^ double mutant. Interestingly, the DEGs between *leg1a*^*zju1/zju1*^ and WT partially reverted to a WT-like pattern in the *upf3a*^*−/−*^*leg1a*^*zju1/zju1*^ double mutant, whereas the DEGs showed a similar pattern between the *leg1a*^*zju1/zju1*^ single and *upf1*^*−/−*^*leg1a*^*zju1/zju1*^ double mutants, further suggesting the important role of Upf3a in the HDGCR. However, whether Upf3a is a general mediator of the HDGCR or is only specific to a limited class of genes, requires further validation via extensive genetic and molecular analysis. Meanwhile, we cannot exclude the possibility that Upf1 may be involved in mediating the HDGCR for a distinct group of genes. Furthermore, although the data from *leg1* mutants is consistent with the induction of the HDGCR, we do not know if it is specifically induced by PTC-bearing mRNA and we cannot rule out the possibility that another class of GCRs could be responsible.

One intriguing question relates to the reason for the difference in the role of Upf3a and Upf1 in the HDGCR process. Our sequencing results provided evidence that Upf3a is not a positive regulator of the NMD pathway. This is consistent with the previous report of Shum et al. implicating mammalian Upf3a as an inhibitor, rather than a positive regulator, of this pathway, probably via competing with Upf3b in the interaction with Upf2^17^. Based on Shum et al.’s hypothesis, we previously proposed that both Upf3a and Upf3b could interact with the complex of PTC-bearing mRNA and the exon junction complex (EJC). When Upf3b forms a complex with Upf2, this complex will be recruited by Upf1 to mediate PTC-bearing mRNA degradation. However, if Upf3a forms the complex with Upf2, it may guide the PTC-bearing mRNA to promote the transcription of compensatory genes^13^. To understand how Upf3a mediates such an HDGCR signal, a future study may be necessary to further identify the Upf3a protein interactomes. Meanwhile, more concrete evidence is required to define the reason for the difference in the role of Upf3a and Upf1 in the HDGCR process. We speculate that the position of the PTC in the mutant mRNA or different auxiliary factors (protein or RNA) associated with the EJC may be important in determining the choice.

H3K4me3 modification at the TSS is often connected to gene activation^34^. Through analyzing the ULI-NChIP-seq data we found that the activation of *leg1b* in the *leg1a*^*zju1/zju1*^ mutant was not coupled with an increase in H3K4me3 modification around the TSS. This finding is discrete from the previous two reports showing an increase of H3K4me3 enrichment around the TSS of the compensatory genes^12,13^. This may suggest that there could be different ways to activate compensatory genes during HDGCR. Cross-comparing the H3K4me3 counts for 70 known liver-enriched genes between the adult and embryonic liver revealed that proportions of these genes are not coupled with an enrichment of H3K4me3 around the TSS, and this phenomenon is even more widespread in the embryonic liver. These observations suggest that, in addition to the H3K4me3 modification around the TSS-site, there might be other transcriptional regulators involved in regulating embryonic liver-enriched genes. 23 genes encoding DNA-binding proteins or transcription regulators were found to be upregulated in the *leg1a*^*zju1/zju1*^ mutant. These genes might serve as candidates for future study for their possible roles in upregulating the expression of *leg1b* in the *leg1a*^*zju1/zju1*^ mutant. Alternatively, other epigenetic modifications could be examined around the *leg1b* locus. However, based on the low FPKM for *leg1b* (~14.6 for *leg1b* vs 198.3 for *leg1a* at 5 dpf), we cannot exclude the possibility that the enrichment of H3K4me3 around the *leg1b* TSS may not be discernable by the current ULI-NChIP-seq analysis.

Genomes usually contain many gene families and the members of each family share different degrees of homology^5,6,9,35,36^. Whether fully or partially, living organisms often take the advantage of redundant functions of homologous genes to cope with genetic mutations and to maintain genetic robustness^2,11^. This clearly represents an important mechanism of GCR. The HDGCR has been applied to explain the observation that ~80% of genetic mutations generated by gene editing approaches do not show an obvious phenotype in zebrafish^11,37^. It is envisaged that homologous genes may co-express within the same cell so that they can compensate for each other’s function when one is deleteriously mutated. However, many homologous genes are differentially expressed in different cell types and the expression of a homologous gene (or genes) has to be activated when the predominant gene is deleteriously mutated, such as was the case for *leg1a* and *leg1b* in this study^21^. The mechanism as to how the compensatory gene is activated remains to be elucidated. Our main finding is that the upregulation of *leg1b* in the *leg1a*^*zju1/zju1*^ mutant is dependent on Upf3a, but not Upf1, and is not coupled with an increase in H3k4me3 around the TSS. This strongly suggests that there might be a complex network or various pathways in either the triggering or regulation of HDGCRs.

## Materials and methods

### Zebrafish lines and maintenance

The zebrafish (*Danio rerio*) AB strain was used as WT in this study. The *leg1a*^*zju1*^ (with 13 bp insertion in the exon 1 of *leg1a*) single, *leg1b*^*zju1*^ (with 14 bp deletion in the exon 2 of *leg1b*) single, and *leg1a*^*zju3*^*leg1b*^*zju1*^ double (*leg1a*^*zju3*^ harboring a 7 bp deletion in *leg1a*) mutant lines were in the AB background and were generated as previously described^21,22^. The *upf3a*^*−/−*^ and *upf1*^*−/−*^ mutant lines were in the Tübingen background and were generated as described^13^. Fish were raised and maintained according to the standard procedures described in ZFIN (http://www.zfin.org).

### Sampling of experiments

More than 5 pairs of parental male and female fish (WT or mutant) were used to set one specific cross. For a cross between homozygous parents, more than 23 embryos were used. For a cross between heterozygous parents, more than 100 embryos were obtained and were genotyped using gene-specific primers (Supplementary Table S22) to obtain the corresponding homozygous mutant embryos as previously described^13,22^. All sets of experiments were repeated at least three times except the ULI-NChIP-seq which was performed with two repeats.

### WISH and organ size measurement

WISH was performed as described previously^38^. Probes were labeled with digoxigenin (DIG). Plasmids containing *fabp10a*, *trypsin,* and *fabp2* gene fragments were obtained and reported previously^38^. The sizes of the liver (marked by the *fabp10a* probe), exocrine pancreas (marked by the *trypsin* probe), or intestinal tube (by the *fabp2* probe), were measured as previously described^20^. In brief, after WISH, liver, exocrine pancrea, or intestinal tube was marked out using their marker genes and then imaged by Nikon AZ100 from left lateral view, after aligning the two eyes of the embryo vertically, or from a dorsal view. The pixels of the mark gene staining of the positive area in each image, as cumulated by the Adobe Photoshop CC 2018 software, were then used as the index of the liver, exocrine pancreas, or intestine sizes, respectively.

### MO injection

MOs were purchased from Gene Tools (Philomath, USA). The *upf3a*-MO, *upf1*-MO, *leg1a*-MO, and *leg1b*-MO were designed as described in previous studies^13,20^. A human *β-globin* (*HBB*) antisense morpholino was used as the standard control (st-MO) for *upf*-MO injection. A 5-base mismatch morpholino (5’-CCATgTCAcACATgTAGCAcGAgTG-3’) was designed as the mismatch control (mismatch-MO) for *leg1*-MO injection. To investigate the mechanism of GCR in *leg1b*^*zju1*^, 1 nL of *upf3a*-MO (0.25 nmol/µL), *upf1*-MO (0.25 nmol/µL), or 1 nL st-MO control (0.25 nmol/µL) was injected into one-cell stage embryos. To investigate the function of *leg1a* or *leg1b* in *leg1b*^*zju1*^ or *leg1a*^*zju1*^, 1 nL *leg1a*-MO (0.25 nmol/µL), *leg1b*-MO (0.25 nmol/µL) or mis-MO was injected into one-cell stage embryos. To test the efficiency of *upf1*-MO and *upf3a*-MO, 1 nL *pcs2* + 5’ UTR:GFP plasmid (80 ng/µL) or 1 nL *upf3a*-MO (0.25 nmol/µL) with 5’ UTR:GFP plasmid (80 ng/µL) or 1 nL *upf1*-MO (0.25 nmol/µL) with 5’ UTR:GFP plasmid (80 ng/µL) was injected into one-cell stage embryos. After 1 day post injection, embryos were collected and imaged using a fluorescence microscope (KEYENCE BZ-X800). The sequences of all MOs are provided in Supplementary Table 22.

### Western blot

Total protein was extracted using an extraction buffer (63 mM Tris-HCl, pH 6.8, 10% glycerol, 5% β-Mercaptoethanol, 3.5% SDS) containing 1× Complete (Roche, 11873580001). Western blot was performed as described previously^20^, using monoclonal antibody against zebrafish Leg1 as the primary antibody. Beta-Tubulin antibody (Cat. #AC021) was purchased from ABclonal company (Wuhan, China).

### qPCR

qPCR was performed as described previously^20^. Total RNA was extracted from WT or *leg1b*^*zju1*^ embryos injected with *st*-MO, *upf3a*-MO, or *upf1*-MO at 1.5 dpf using a TRIZOL reagent (AidLab). For qPCR, total RNA was treated with DNaseI before reverse transcription and purified using an RNA clean kit (AidLab). qPCR was performed in a CFX96TM Real-Time System (Bio-Rad) using a C1000 Thermal Cycle (Bio-Rad) according to the manufacturer’s instructions. Total RNA was normalized to the zebrafish *actb1* gene. The primer pairs used are listed in Supplementary Table 22.

### RNA-seq analysis

As stated in the text, total RNA was extracted from the embryos of specific genotypes using a TRIZOL reagent (AidLab) according to the manufacturer’s protocol. Three independent RNA samples were obtained for each genotype in each set of RNA-seq analysis. Isolation of mRNA, library construction, high throughput sequencing, and data filtering to obtain clean reads, were performed by Novogene company (Beijing, China). Clean reads were mapped to the zebrafish genome (Danio_rerio.GRCz11) with Hisat2 (v 2.2.1)^39^. FPKM was obtained for each gene in the RNA-seq data using the feature Counts (v 2.0.1) package^40^. DEGs were obtained using DESeq2 R package (v 1.30.1)^41^.

### ULI-NChIP-seq

ChIP was performed using the ULI-NChIP protocol as described previously^42^ with 150 livers micro-dissected from the WT, *leg1a*^*zju1/zju1*^ single, *upf3a*^*−/−*^ single, and *upf3a*^*−/−*^*leg1a*^*zju1/zju1*^ double mutant embryos at 5 dpf. Two independent ChIP samples were obtained for each genotype in each set of ULI-NChIP-seq analyses. After washing with cold PBS, liver cells were lysed in cell lysis buffer (10 mM Tris-HCl, pH 7.5, 10 mM NaCl, 0.5% NP-40, 1× EDTA-free protease inhibitor cocktail, and 1 mM phenylmethanesulfonyl fluoride (Sigma) on ice for 10 min. The nuclei were pelleted at 3500 rpm for 5 min at 4 °C, and then re-suspended in nuclear extraction buffer (10 mM Tris-HCl, pH 8.0, 140 mM NaCl, 5 mM MgCl, 0.6% NP-40, 1× EDTA-free protease inhibitor cocktail and 1 mM phenylmethanesulfonyl fluoride on ice for 15 min. Chromatin was fragmented by using MNase (NEB, Cat. #M0247S) (final concentration 4U/µL) for 5 min at 37 °C. The reaction was stopped by adding 16.7 mM EDTA, 8.4 mM EGTA, 1% Triton X-100, and 1% sodium deoxycholate. Fragmented chromatin was diluted in Complete immunoprecipitation buffer (20 mM Tris-HCl, pH 8.0, 2 mM EDTA, 150 mM NaCl, 0.1% Triton X-100, 1× EDTA-free protease inhibitor cocktail and 1 mM phenylmethanesulfonyl fluoride). Chromatin was pre-cleared with 5 µL of 1:1 Protein A + G Magnetic Beads (Sigma, Cat. #16-663) and IPed with 2 μg of H3K4me3 antibody (Abcam, Cat. #ab8580)-beads complexes overnight at 4 °C with rotation. IPed complexes were washed twice with 150 µL of low salt wash buffer (20 mM Tris-HCl, pH 8.0, 2 mM EDTA, 150 mM NaCl, 1% Triton X-100, 0.1% SDS, 1× EDTA-free protease inhibitor cocktail and 1 mM phenylmethanesulfonyl fluoride) and twice with 150 µL of high salt wash buffer (20 mM Tris-HCl, pH 8.0, 2 mM EDTA, 500 mM NaCl, 1% Triton X-100, 0.1% SDS, 1× EDTA-free protease inhibitor cocktail and 1 mM phenylmethanesulfonyl fluoride). The bead-bound DNA was then eluted using ChIP elution buffer (100 mM NaHCO_3_ and 1% SDS) for 3 h at 68 °C. After treatment with proteinase K and RNase A, eluted chromatin was purified using phenol-chloroform extraction, and raw ChIP material was re-suspended in 10 mM Tris-HCl, at pH 8.0. Library construction, high throughput sequencing, and data filtering to get the clean reads were performed by the Anoroad company. Clean reads were mapped to the zebrafish genome (Danio_rerio.GRCz11) using the BWA (v 0.7.17)^43^. Regions of H3K4me3 enrichment over background were identified by using the MACS2 (v 2.2.7.1) peak calling software^44^. Normalized read distribution profiles were obtained using Deeptools (v 3.2.1)^45^.

### Statistical analysis

Student’s *t*-tests were used for statistical comparisons (*P < 0.05; **P < 0.01; ***P < 0.001; ns, no significant difference).

## Supplementary information

Supplementary Figures S1-S9

Table S1

Table S2

Table S3

Table S4

Table S5

Table S6

Table S7

Table S8

Table S9

Table S10

Table S11

Table S12

Table S13

Table S14

Tables S15

Table S16

Table S17

Table S18

Table S19

Table S20

Table S21

Table S22

## Data Availability

All relevant data are available from the authors and/or included in the manuscript or supplementary information. RNA-seq data and ULI-NChIP-seq data in this paper have been deposited in the NCBI database (BioProject: PRJNA874604).
