# Supplementary material for: Upf3a but not Upf1 mediates the genetic compensation response induced by leg1 deleterious mutations in an H3K4me3-independent manner
Source: Cell Discov. 2023 Jun 27;9:63. doi: 10.1038/s41421-023-00550-2 (PMC10300044; doi:10.1038/s41421-023-00550-2)
Supplement: Supplementary file 1 — Supplementary Figures S1-S9 [file 41421_2023_550_MOESM1_ESM.pdf]

Fig.S1

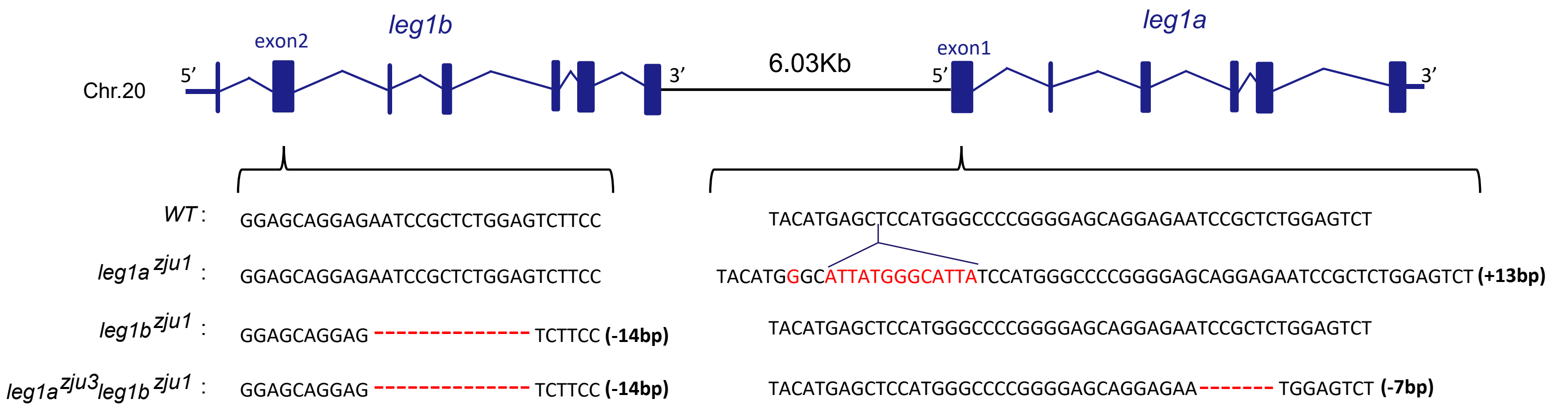

leg1b
leg1a
exon2
exon1
Chr.20
5′
3′
6.03Kb
WT :
leg1a zju1 :
leg1b zju1 :
leg1a zju3 leg1b zju1 :
GGAGCAGGAGAATCCGCTCTGGAGTCTTCC
TACATGAGCTCCATGGGCCCCGGGGAGCAGGAGAATCCGCTCTGGAGTCT
GGAGCAGGAGAATCCGCTCTGGAGTCTTCC
TACATGGGCATTATGGGCATTATCCATGGGCCCCGGGGAGCAGGAGAATCCGCTCTGGAGTCT (+13bp)
GGAGCAGGAG TCTTCC (-14bp)
TACATGAGCTCCATGGGCCCCGGGGAGCAGGAGAATCCGCTCTGGAGTCT
GGAGCAGGAG TCTTCC (-14bp)
TACATGAGCTCCATGGGCCCCGGGGAGCAGGAGAA TGGAGTCT (-7bp)

Fig. S1 ***leg1a*** $^{zju3/zju3}$ ***leg1b*** $^{zju1/zju1}$ **double mutant exhibits a smaller liver, exocrine pancreas and intestinal tube phenotype.** Diagram depicting the mutations carried by *leg1a* $^{zju1/zju1}$ single, *leg1b* $^{zju1/zju1}$ single and *leg1a* $^{zju3/zju3}$ *leg1b* $^{zju1/zju1}$ double mutants. Top panels, showing the genomic structures of *leg1a* and *leg1b* which are linked by a 6.03kb genomic DNA fragment on chromosome 20. Lower panels, showing the WT *leg1a* and *leg1b* sequences where mutations occurred in each mutant. Red letters, inserted nucleotides; red dashed line, deleted nucleotides. The number of altered nucleotides (bp) is provided in the bracket.

Fig.S2

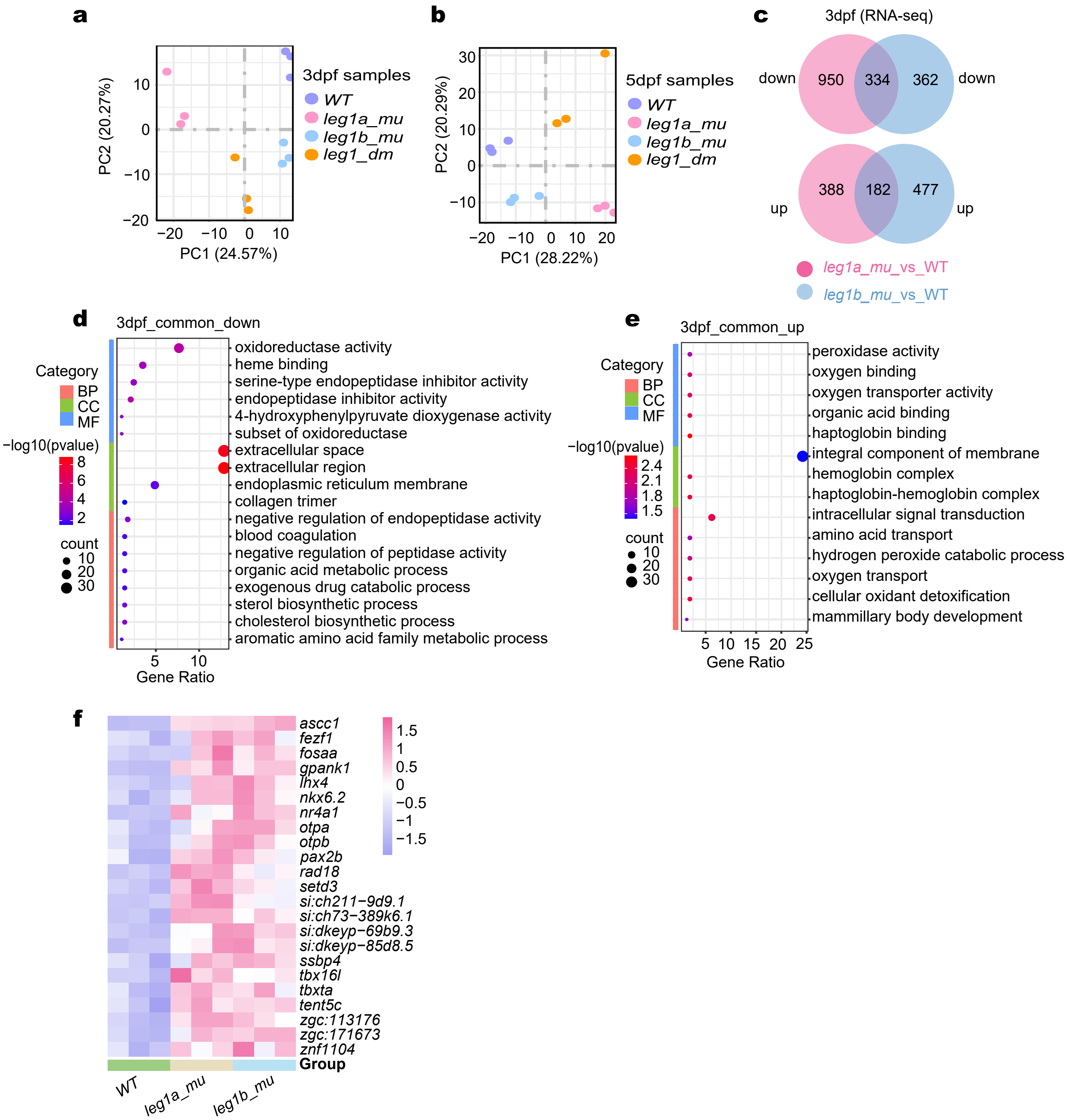
a
PC2 (20.27%)
PC1 (24.57%)
3dpf samples
WT
leg1a_mu
leg1b_mu
leg1_dm
b
PC2 (20.29%)
PC1 (28.22%)
5dpf samples
WT
leg1a_mu
leg1b_mu
leg1_dm
c
3dpf (RNA-seq)
down
950
334
362
down
388
182
477
up
up
leg1a_mu_vs_WT
leg1b_mu_vs_WT
d
3dpf_common_down
Category
BP
CC
MF
−log10(pvalue)
count
oxidoreductase activity
heme binding
serine-type endopeptidase inhibitor activity
endopeptidase inhibitor activity
4-hydroxyphenylpyruvate dioxygenase activity
subset of oxidoreductase
extracellular space
extracellular region
endoplasmic reticulum membrane
collagen trimer
negative regulation of endopeptidase activity
blood coagulation
negative regulation of peptidase activity
organic acid metabolic process
exogenous drug catabolic process
sterol biosynthetic process
cholesterol biosynthetic process
aromatic amino acid family metabolic process
Gene Ratio
e
3dpf_common_up
Category
BP
CC
MF
−log10(pvalue)
count
peroxidase activity
oxygen binding
oxygen transporter activity
organic acid binding
haptoglobin binding
integral component of membrane
hemoglobin complex
haptoglobin-hemoglobin complex
intracellular signal transduction
amino acid transport
hydrogen peroxide catabolic process
oxygen transport
cellular oxidant detoxification
mammillary body development
Gene Ratio
f
ascc1
fezf1
fosaa
gpank1
lhx4
nkx6.2
nr4a1
otpa
otpb
pax2b
rad18
setd3
si:ch211−9d9.1
si:ch73−389k6.1
si:dkeyp−69b9.3
si:dkeyp−85d8.5
ssbp4
tbx16l
tbxta
tent5c
zgc:113176
zgc:171673
znf1104
Group
WT
leg1a_mu
leg1b_mu

Fig. S2 Cross-comparison of the DEGs identified in *leg1a*$^{zju1/zju1}$ and *leg1b*$^{zju1/zju1}$ single mutants. a, b PCA analysis of RNA-seq data for WT, *leg1a*$^{zju1/zju1}$ (*leg1a_mu*) single, *leg1b*$^{zju1/zju1}$ (*leg1b_mu*) single and *leg1a*$^{zju3/zju3}$ *leg1b*$^{zju1/zju1}$ (*leg1_dm*) double mutants at 3dpf (**a**) and 5dpf (**b**), respectively. Three independent samples for each genotype were analyzed. **c** Venn maps showing the number of distinct and shared downregulated (top panel) and upregulated (bottom panel) DEGs in *leg1a_mu* single and *leg1b_mu* single mutants at 3dpf. **d, e** GO analysis of the shared 334 downregulated and 182 upregulated DEGs between *leg1a_mu* single and *leg1b_mu* single mutants. **f** Heatmap showing the 23 shared upregulated DEGs in *leg1a_mu* single and *leg1b_mu* single mutants. These 23 genes encode products related to DNA-binding or transcription regulation.

Fig. S3

a

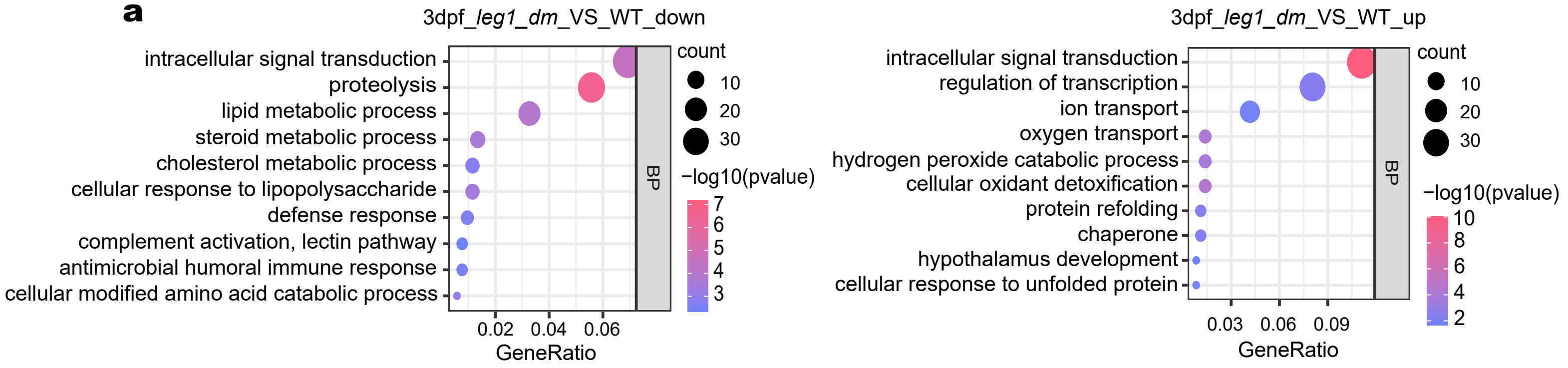

b

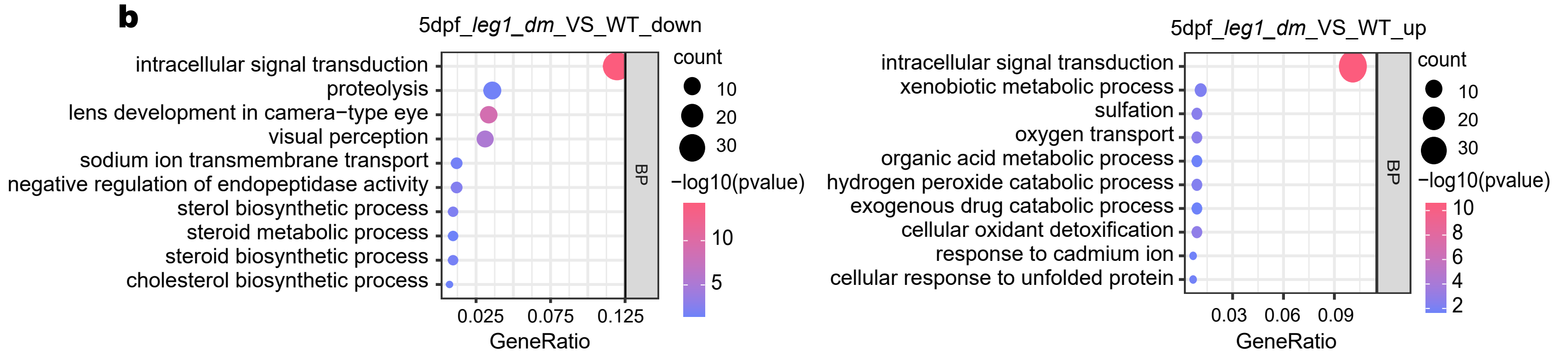

c

intracellular signal transduction

3dpf down_DEGs		5dpf down_DEGs	
Gene Symbol	Ensembl Gene ID	Ensembl Gene ID	Gene Symbol
CABZ01072815.1	ENSDARG00000104770	ENSDARG00000104770	CABZ01072815.1
si:ch211-191a16.5	ENSDARG00000096545	ENSDARG00000096545	si:ch211-191a16.5
si:dkey-190j3.2	ENSDARG00000099152	ENSDARG00000099152	si:dkey-190j3.2
si:ch211-201o1.1	ENSDARG00000088689	ENSDARG00000088689	si:ch211-201o1.1
si:dkey-207m2.4	ENSDARG00000100654	ENSDARG00000100654	si:dkey-207m2.4
si:ch211-266c8.1	ENSDARG00000103844	ENSDARG00000103844	si:ch211-266c8.1
CR854897.1	ENSDARG00000095528	ENSDARG00000095528	CR854897.1
CABZ01074397.1	ENSDARG00000104672	ENSDARG00000104672	CABZ01074397.1
si:ch211-59d8.3	ENSDARG00000098514	ENSDARG00000098514	si:ch211-59d8.3
si:dkey-238k10.2	ENSDARG00000104380	ENSDARG00000104380	si:dkey-238k10.2
si:dkey-238k10.1	ENSDARG00000099384	ENSDARG00000099384	si:dkey-238k10.1
si:ch73-168d20.1	ENSDARG00000039900	ENSDARG00000039900	si:ch73-168d20.1
nlrc8	ENSDARG00000076819	ENSDARG00000076819	nlrc8
si:dkeyp-4c4.1	ENSDARG00000103755	ENSDARG00000103755	si:dkeyp-4c4.1
si:ch211-233m11.1	ENSDARG00000074653	ENSDARG00000074653	si:ch211-233m11.1
si:dkey-121n8.7	ENSDARG00000094067	ENSDARG00000094067	si:dkey-121n8.7
CABZ01029938.1	ENSDARG00000099006	ENSDARG00000099006	CABZ01029938.1
si:dkeyp-82b4.2	ENSDARG00000112648	ENSDARG00000112648	si:dkeyp-82b4.2
si:ch211-194e1.7	ENSDARG00000096449	ENSDARG00000096449	si:ch211-194e1.7
si:ch211-181d7.3	ENSDARG00000068621	ENSDARG00000068621	si:ch211-181d7.3
si:dkey-238k10.3	ENSDARG00000101992	ENSDARG00000101992	si:dkey-238k10.3
si:dkey-28k24.2	ENSDARG00000110878	ENSDARG00000102490	asb2b
si:dkeyp-51g9.4	ENSDARG00000099899	ENSDARG00000027724	dgkb
si:ch211-236p5.3	ENSDARG00000086418	ENSDARG00000101484	si:dkey-264f17.2
si:ch211-226o13.1	ENSDARG00000087714	ENSDARG00000098179	si:dkeyp-86c4.1
AL929028.8	ENSDARG00000105095	ENSDARG00000093024	si:ch211-213a13.2
si:ch73-286h23.4	ENSDARG00000090193	ENSDARG00000110142	CABZ01113818.1
		ENSDARG00000086179	si:dkey-3h2.4
		ENSDARG00000103281	si:dkey-56m15.9
		ENSDARG00000113660	si:ch211-196f19.1
		ENSDARG00000101728	si:dkeyp-80d11.12
		ENSDARG00000104341	si:ch211-76m11.8
		ENSDARG00000068127	si:ch211-194e1.9
		ENSDARG00000075949	prkd1

d

intracellular signal transduction

3dpf up_DEGs		5dpf up_DEGs	
Gene Symbol	Ensembl Gene ID	Ensembl Gene ID	Gene Symbol
CABZ01074307.1	ENSDARG00000099775	ENSDARG00000099775	CABZ01074307.1
si:ch211-196f19.1	ENSDARG00000103381	ENSDARG00000103381	si:ch211-196f19.1
si:dkey-159n16.2	ENSDARG00000114577	ENSDARG00000114577	si:dkey-159n16.2
asb14b	ENSDARG00000087741	ENSDARG00000087741	asb14b
si:dkey-264f17.1	ENSDARG00000100219	ENSDARG00000100219	si:dkey-264f17.1
si:dkey-11n14.1	ENSDARG00000101374	ENSDARG00000101374	si:dkey-11n14.1
si:dkey-31n13.4	ENSDARG00000101312	ENSDARG00000101312	si:dkey-31n13.4
si:ch211-215p11.1	ENSDARG00000101598	ENSDARG00000101598	si:ch211-215p11.1
si:ch211-214b16.4	ENSDARG00000074345	ENSDARG00000074345	si:ch211-214b16.4
si:dkey-105i14.1	ENSDARG00000077697	ENSDARG00000077697	si:dkey-105i14.1
si:ch211-197e7.1	ENSDARG00000095200	ENSDARG00000095200	si:ch211-197e7.1
CABZ01049625.1	ENSDARG00000116043	ENSDARG00000116043	CABZ01049625.1
si:ch211-76m11.8	ENSDARG00000098390	ENSDARG00000098390	si:ch211-76m11.8
CABZ01009512.2	ENSDARG00000104424	ENSDARG00000104424	CABZ01009512.2
si:ch211-214b16.2	ENSDARG00000102593	ENSDARG00000102593	si:ch211-214b16.2
prkcg	ENSDARG00000004561	ENSDARG00000055934	si:ch1073-416j23.1
FP236318.3	ENSDARG00000110485	ENSDARG00000105063	si:ch211-64i20.3
si:dkey-238o14.3	ENSDARG00000098077	ENSDARG00000091013	si:dkey-84h14.2
si:dkey-61p9.9	ENSDARG00000092759	ENSDARG00000093402	si:dkey-286j17.4
si:ch211-227e10.1	ENSDARG00000104968	ENSDARG00000099984	CABZ01023255.1
si:ch211-215p11.3	ENSDARG00000101450	ENSDARG00000113896	si:dkey-29p23.1
map4k6	ENSDARG00000033444	ENSDARG00000103955	si:dkey-29j8.1
rgs11	ENSDARG00000037646	ENSDARG00000077068	prkcg
si:ch211-42i6.2	ENSDARG00000086130	ENSDARG00000101916	si:dkeyp-44d3.5
		ENSDARG00000090160	zgc:173709
		ENSDARG00000101754	BX664622.1

Fig. S3 GO analysis of DEGs revealed that the 'intracellular signal transduction' process is the most significant process altered in *leg1a*$^{zju3/zju3}$ *leg1b*$^{zju1/zju1}$ double mutants. **a, b** GO biological process (GO_BP) analysis of the down- and up-regulated DEGs identified in the *leg1a*$^{zju3/zju3}$ *leg1b*$^{zju1/zju1}$ double mutants at 3dpf (**a**) and 5dpf (**b**), respectively. Top10 categories are shown. **c, d** Comparison of the down-regulated (**c**) and up-regulated (**d**) DEGs classified into the 'intracellular signal transduction' categories at 3dpf and 5dpf, respectively. The down-regulated DEGs at 3dpf and 5dpf shared 21 genes (**c**) while the up-regulated genes at 3dpf and 5dpf shared 15 genes (**d**). Note that majority of these genes have not been assigned with a genuine name.

Fig. S4

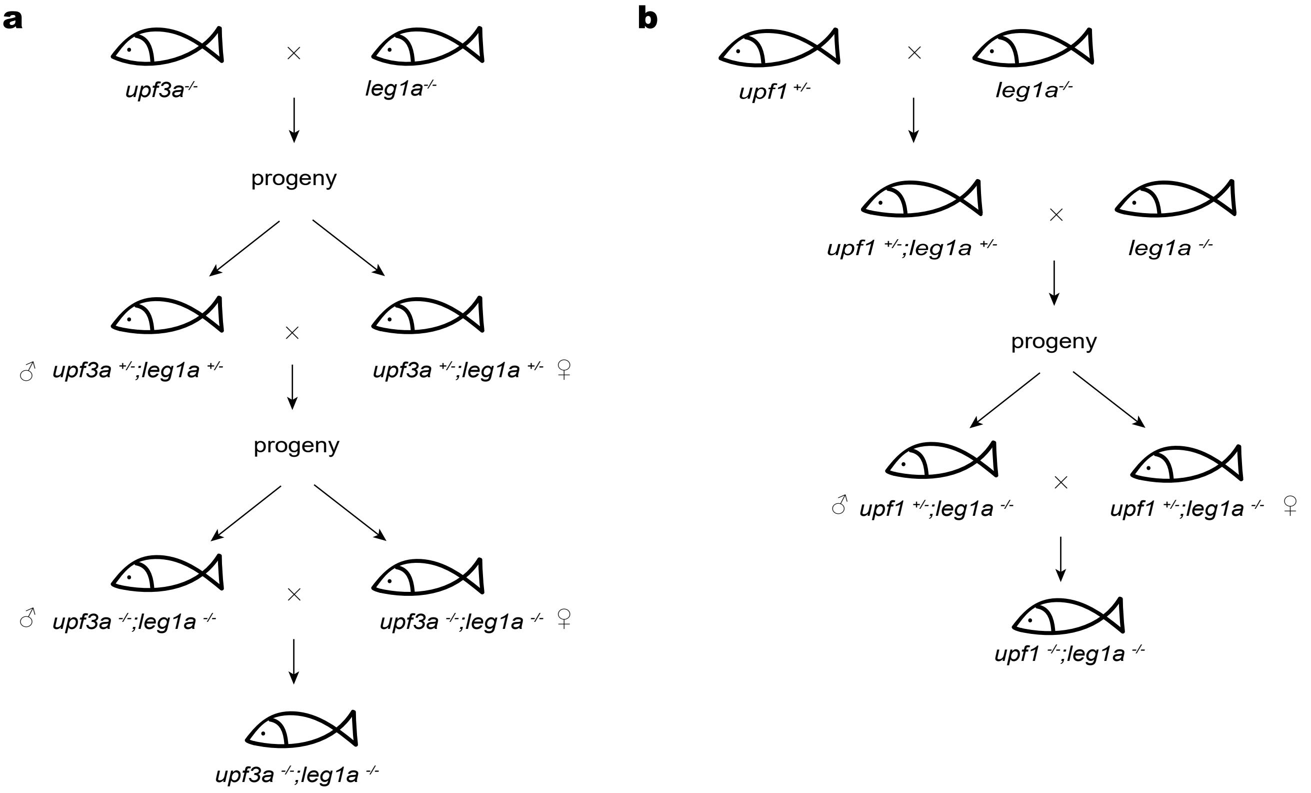
a
upf3a-/-
×
leg1a-/-
progeny
♂ upf3a +/-;leg1a +/-
×
upf3a +/-;leg1a +/- ♀
progeny
♂ upf3a -/-;leg1a -/-
×
upf3a -/-;leg1a -/- ♀
upf3a -/-;leg1a -/-
b
upf1 +/-
×
leg1a-/-
upf1 +/-;leg1a +/-
×
leg1a -/-
progeny
♂ upf1 +/-;leg1a -/-
×
upf1 +/-;leg1a -/- ♀
upf1 -/-;leg1a -/-

Fig. S4 Phenotypic analysis of the *upf3a*$^{-/-}$ *leg1a*$^{zju1/zju1}$ and *upf1*$^{-/-}$ *leg1a*$^{zju1/zju1}$ double mutants. a, b Flow chart showing the approach for generating the *upf3a*$^{-/-}$ *leg1a*$^{zju1/zju1}$ and *upf1*$^{-/-}$ *leg1a*$^{zju1/zju1}$ double mutants.

Fig. S5

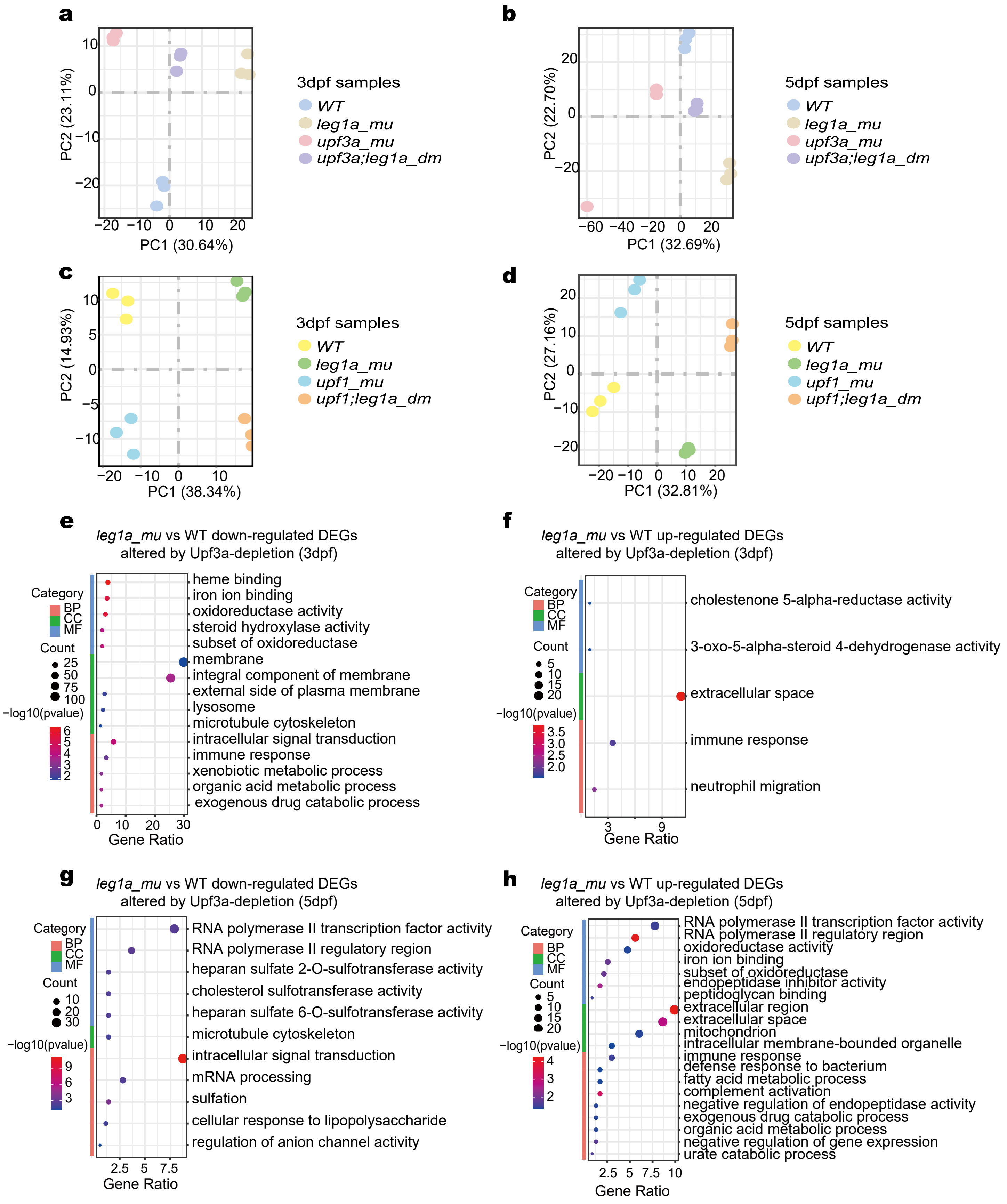
a
3dpf samples
WT
leg1a_mu
upf3a_mu
upf3a;leg1a_dm
PC1 (30.64%)
PC2 (23.11%)
b
5dpf samples
WT
leg1a_mu
upf3a_mu
upf3a;leg1a_dm
PC1 (32.69%)
PC2 (22.70%)
c
3dpf samples
WT
leg1a_mu
upf1_mu
upf1;leg1a_dm
PC1 (38.34%)
PC2 (14.93%)
d
5dpf samples
WT
leg1a_mu
upf1_mu
upf1;leg1a_dm
PC1 (32.81%)
PC2 (27.16%)
e
leg1a_mu vs WT down-regulated DEGs
altered by Upf3a-depletion (3dpf)
Category
BP
CC
MF
Count
−log10(pvalue)
heme binding
iron ion binding
oxidoreductase activity
steroid hydroxylase activity
subset of oxidoreductase
membrane
integral component of membrane
external side of plasma membrane
lysosome
microtubule cytoskeleton
intracellular signal transduction
immune response
xenobiotic metabolic process
organic acid metabolic process
exogenous drug catabolic process
Gene Ratio
f
leg1a_mu vs WT up-regulated DEGs
altered by Upf3a-depletion (3dpf)
Category
BP
CC
MF
Count
−log10(pvalue)
cholestenone 5-alpha-reductase activity
3-oxo-5-alpha-steroid 4-dehydrogenase activity
extracellular space
immune response
neutrophil migration
Gene Ratio
g
leg1a_mu vs WT down-regulated DEGs
altered by Upf3a-depletion (5dpf)
Category
BP
CC
MF
Count
−log10(pvalue)
RNA polymerase II transcription factor activity
RNA polymerase II regulatory region
heparan sulfate 2-O-sulfotransferase activity
cholesterol sulfotransferase activity
heparan sulfate 6-O-sulfotransferase activity
microtubule cytoskeleton
intracellular signal transduction
mRNA processing
sulfation
cellular response to lipopolysaccharide
regulation of anion channel activity
Gene Ratio
h
leg1a_mu vs WT up-regulated DEGs
altered by Upf3a-depletion (5dpf)
Category
BP
CC
MF
Count
−log10(pvalue)
RNA polymerase II transcription factor activity
RNA polymerase II regulatory region
oxidoreductase activity
iron ion binding
subset of oxidoreductase
endopeptidase inhibitor activity
peptidoglycan binding
extracellular region
extracellular space
mitochondrion
intracellular membrane-bounded organelle
immune response
defense response to bacterium
fatty acid metabolic process
complement activation
negative regulation of endopeptidase activity
exogenous drug catabolic process
organic acid metabolic process
negative regulation of gene expression
urate catabolic process
Gene Ratio

Fig. S5 RNA-seq analysis of the *leg1a* and *leg1b* transcripts in the *upf3a*$^{-/-}$ *leg1a*$^{zju1/zju1}$ and *upf1*$^{-/-}$ *leg1a*$^{zju1/zju1}$ double mutants. a, b PCA analysis of RNA-seq data for WT, *leg1a*$^{zju1/zju1}$ (*leg1a_mu*) single, *upf3a*$^{-/-}$ (*upf3a_mu*) single and *upf3a*$^{-/-}$ *leg1a*$^{zju1/zju1}$ (*upf3a;leg1a_dm*) double mutants at 3dpf (**a**) and 5dpf (**b**), respectively. Three independent samples for each genotype were analyzed. Due to the obvious derivation of one *ufp3a_mu* sample at 5dpf (**b**), we only used the RNA-seq data from two *ufp3a_mu* samples in our analysis. **c, d** PCA analysis of RNA-seq data for WT, *leg1a*$^{zju1/zju1}$ (*leg1a_mu*) single, *upf1*$^{-/-}$ (*upf1_mu*) single and *upf1*$^{-/-}$ *leg1a*$^{zju1/zju1}$ (*upf1;leg1a_dm*) double mutants at 3dpf (**c**) and 5dpf (**d**), respectively. Three independent samples for each genotype were analyzed. **e-h** GO analysis of the *leg1a*$^{zju1/zju1}$ downregulated (**e, g**) and upregulated (**f, h**) DEGs altered by the Upf3a-depletion at 3dpf (**e, f**) and 5dpf (**g, h**), respectively.

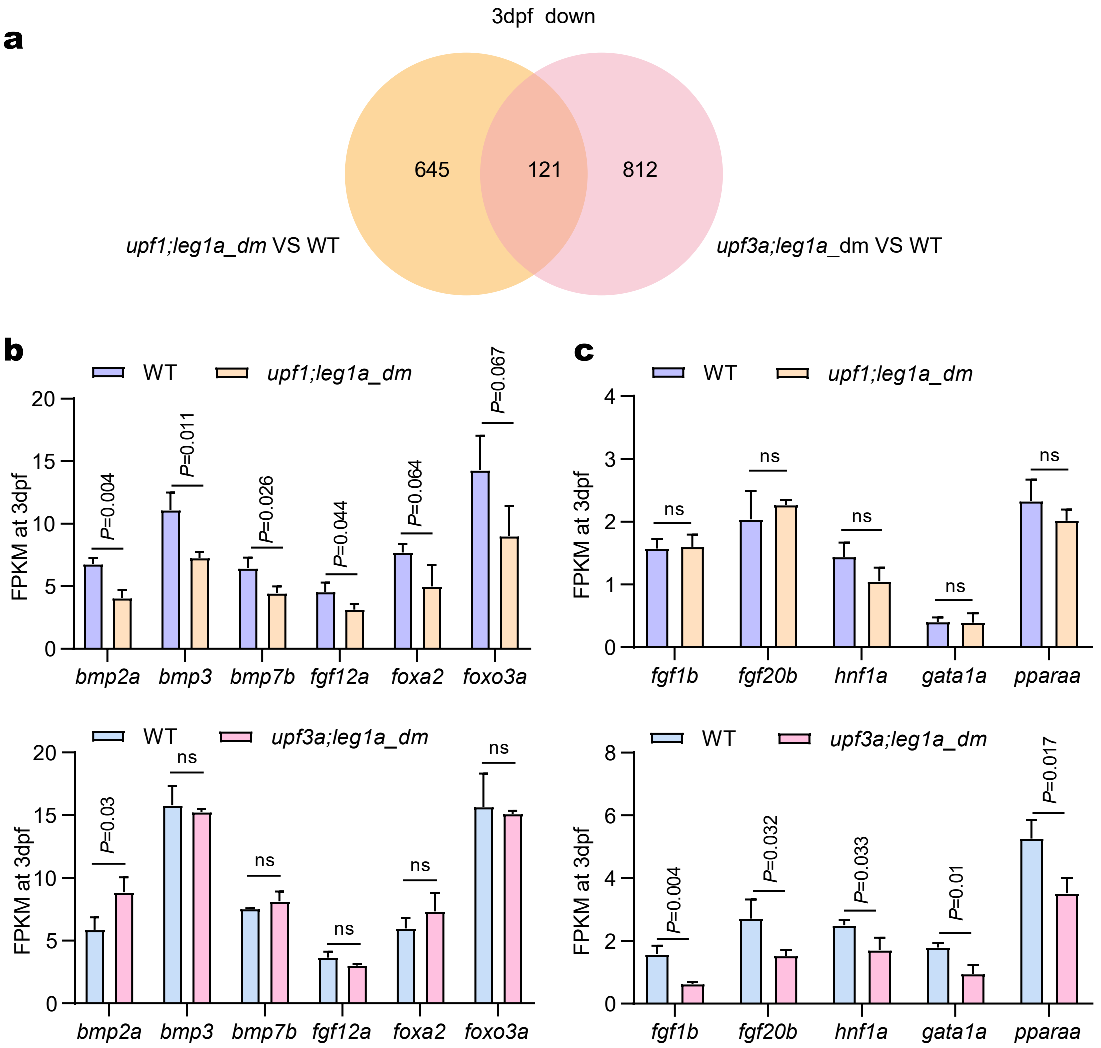
a
3dpf down
645
121
812
upf1;leg1a_dm VS WT
upf3a;leg1a_dm VS WT
b
WT
upf1;leg1a_dm
FPKM at 3dpf
P=0.004
P=0.011
P=0.026
P=0.044
P=0.064
P=0.067
bmp2a bmp3 bmp7b fgf12a foxa2 foxo3a
c
fgf1b fgf20b hnf1a gata1a pparaa
ns
upf3a;leg1a_dm
P=0.03
P=0.004
P=0.032
P=0.033
P=0.01
P=0.017

Fig. S6 The small liver phenotype observed in *upf3a*$^{-/-}$ *leg1a*$^{zju1/zju1}$ and *upf1*$^{-/-}$ *leg1a*$^{zju1/zju1}$ double mutants might be caused by the downregulation of distinct genes involved in regulating liver development. a Venn maps showing the number of distinct and shared downregulated DEGs in *upf1*$^{-/-}$ *leg1a*$^{zju1/zju1}$ (*upf1;leg1a_dm*) double mutants and *upf3a*$^{-/-}$ *leg1a*$^{zju1/zju1}$ (*upf3a;leg1a_dm*) double mutants at 3dpf. **b, c** Histogram showing the statistical analysis of transcript counts (FPKM) from the RNA-seq data of the genes which were downregulated in *upf1*$^{-/-}$ *leg1a*$^{zju1/zju1}$ (**b**, upper panels) but not so in *upf3a*$^{-/-}$ *leg1a*$^{zju1/zju1}$ (**b**, lower panel), or downregulated in *upf3a*$^{-/-}$ *leg1a*$^{zju1/zju1}$ (**c**, lower panel) but not so in *upf1*$^{-/-}$ *leg1a*$^{zju1/zju1}$ (**c**, upper panel) at 3dpf. These genes are presumably related to the liver development. obtained from WT, and embryos at 3dpf. Statistic *p*-value was provided for each of the pair comparison. ns, no significant difference.

Fig. S7

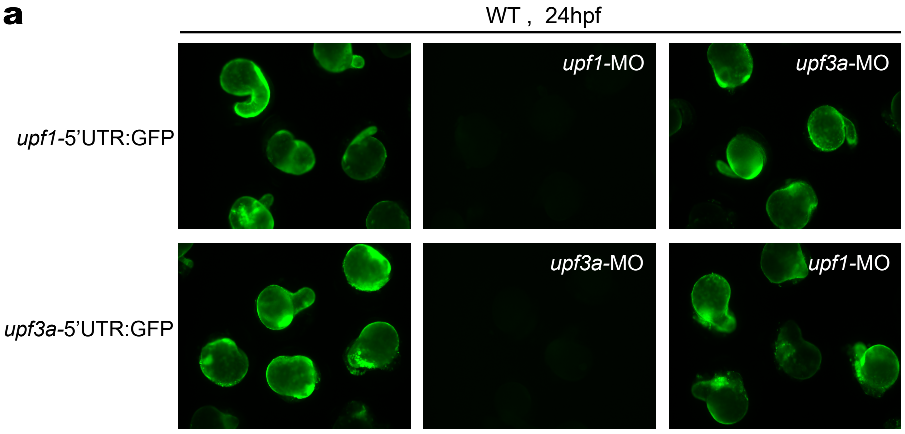
a
WT， 24hpf
upf1-5’UTR:GFP
upf3a-5’UTR:GFP
upf1-MO
upf3a-MO
upf3a-MO
upf1-MO

Fig. S7 Verification of the efficiency of the *upf1*-MO and *upf3a*-MO. The 5'UTR sequences of the *upf1* and *upf3a* cDNA was cloned upstream to the reporter gene *egfp*, respectively. The plasmids were co-injected with the *upf1*-MO or *upf3a*-MO. The Egfp fluorescence was visualized under a fluorescence microscope (KEYENCE BZ-X800).

Fig. S8

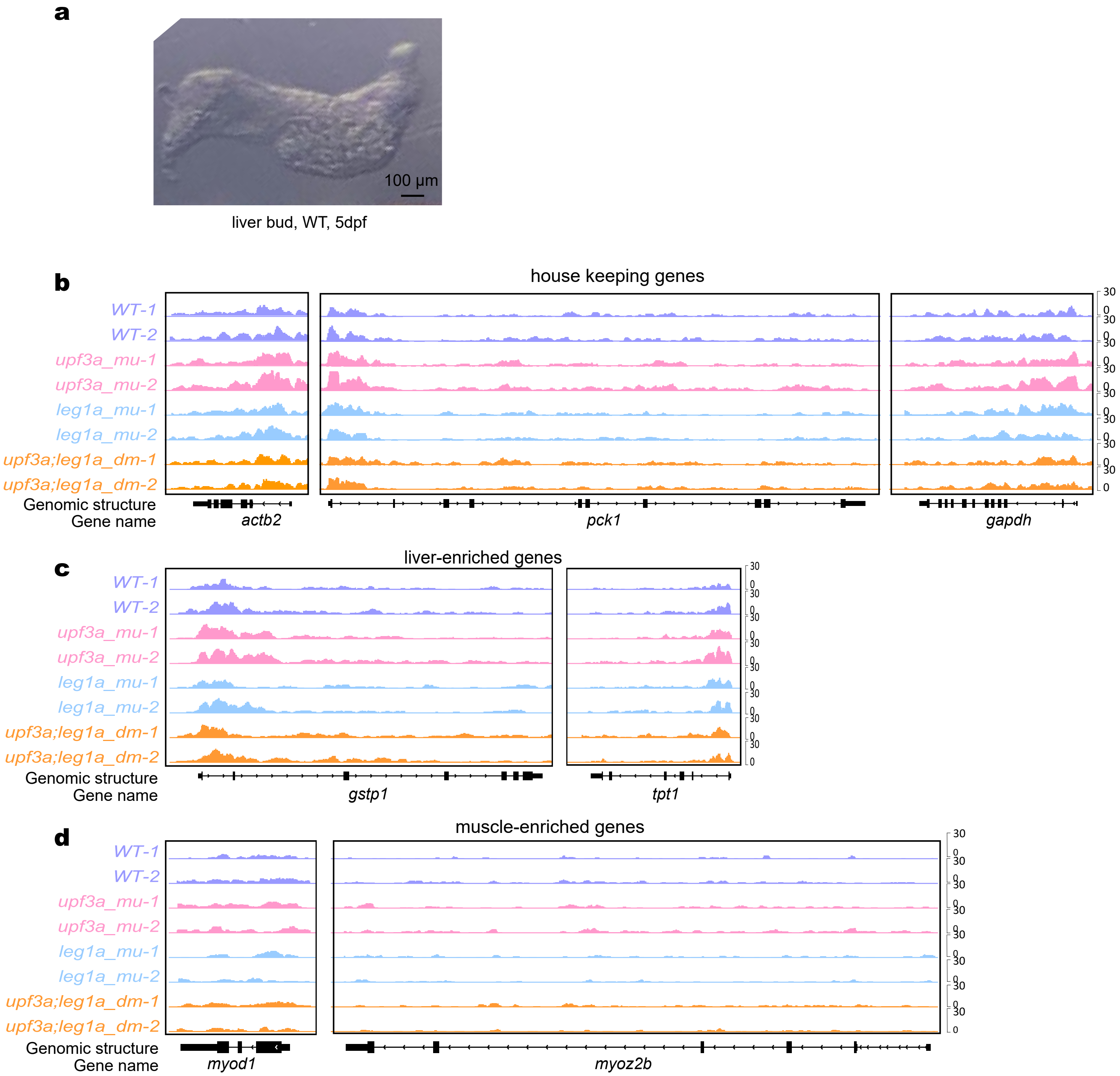

a
100 µm
liver bud, WT, 5dpf
b
house keeping genes
WT-1
WT-2
upf3a_mu-1
upf3a_mu-2
leg1a_mu-1
leg1a_mu-2
upf3a;leg1a_dm-1
upf3a;leg1a_dm-2
Genomic structure
Gene name
actb2
pck1
gapdh
30
0
c
liver-enriched genes
WT-1
WT-2
upf3a_mu-1
upf3a_mu-2
leg1a_mu-1
leg1a_mu-2
upf3a;leg1a_dm-1
upf3a;leg1a_dm-2
Genomic structure
Gene name
gstp1
tpt1
d
muscle-enriched genes
WT-1
WT-2
upf3a_mu-1
upf3a_mu-2
leg1a_mu-1
leg1a_mu-2
upf3a;leg1a_dm-1
upf3a;leg1a_dm-2
Genomic structure
Gene name
myod1
myoz2b

Fig. S8 ULI-NChIP-seq analysis of the H3K4me3 enrichment. a An example of the micro-dissected liver buds from 5dpf WT embryos. **b-d** Genome browser view showing the enrichment of H3K4me3 in the genomic region of representative house keeping genes (**b**), liver-enriched genes (**c**) and muscle-enriched genes (**d**) in WT, *leg1a*$^{zju1/zju1}$ (*leg1a_mu*) single, *upf3a*$^{-/-}$ (*upf3a_mu*) single and *upf3a*$^{-/-}$ *leg1a*$^{zju1/zju1}$ (*upf3a;leg1a_dm*) double mutant embryos at 5dpf.

Fig. S9-1

a

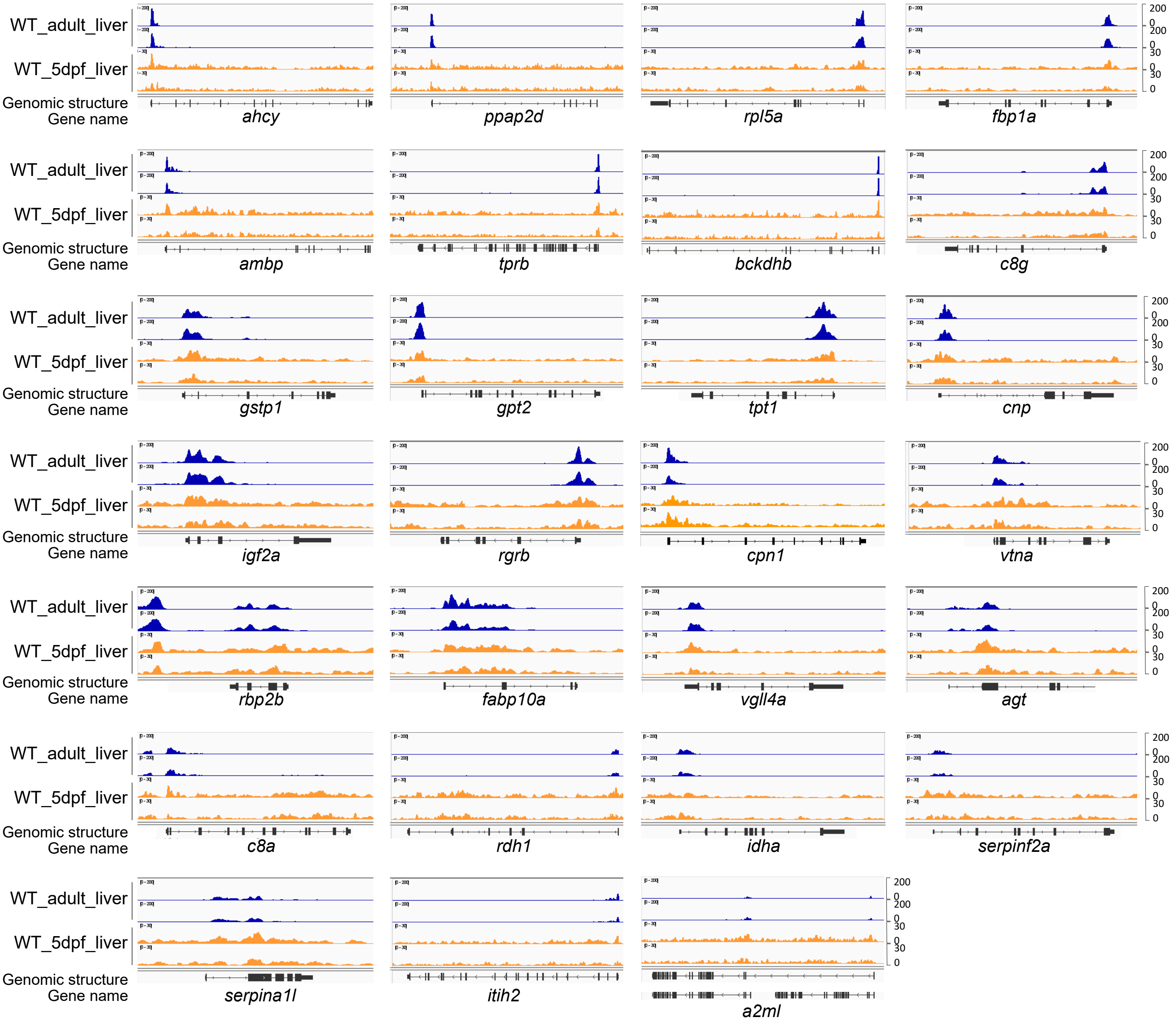

WT_adult_liver
WT_5dpf_liver
Genomic structure
Gene name
ahcy
ppap2d
rpl5a
fbp1a
ambp
tprb
bckdhb
c8g
gstp1
gpt2
tpt1
cnp
igf2a
rgrb
cpn1
vtna
rbp2b
fabp10a
vgll4a
agt
c8a
rdh1
idha
serpinf2a
serpina1l
itih2
a2ml
200
0
30

b

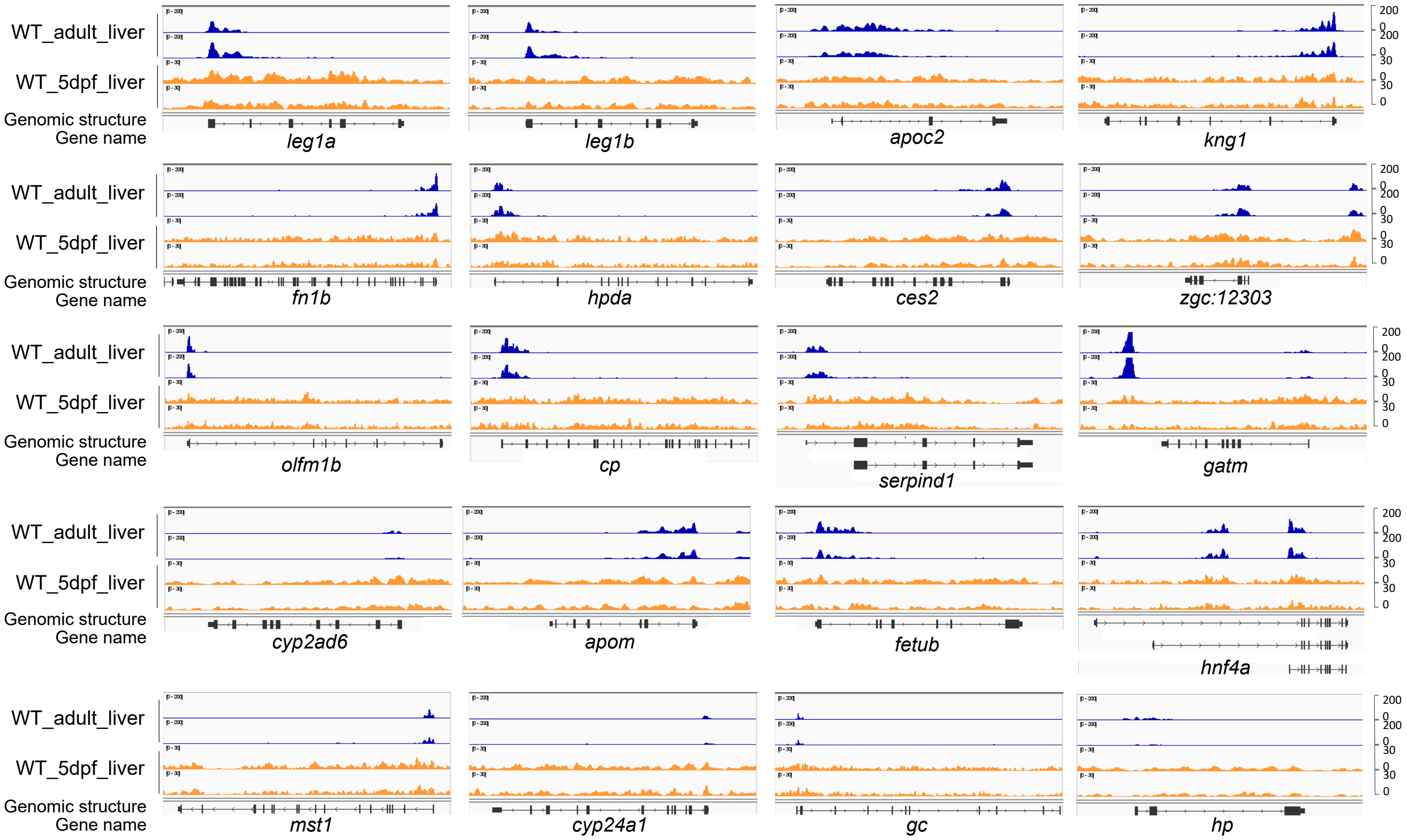
WT_adult_liver
WT_5dpf_liver
Genomic structure
Gene name
leg1a
leg1b
apoc2
kng1
fn1b
hpda
ces2
zgc:12303
olfm1b
cp
serpind1
gatm
cyp2ad6
apom
fetub
hnf4a
mst1
cyp24a1
gc
hp
200
0
30

c

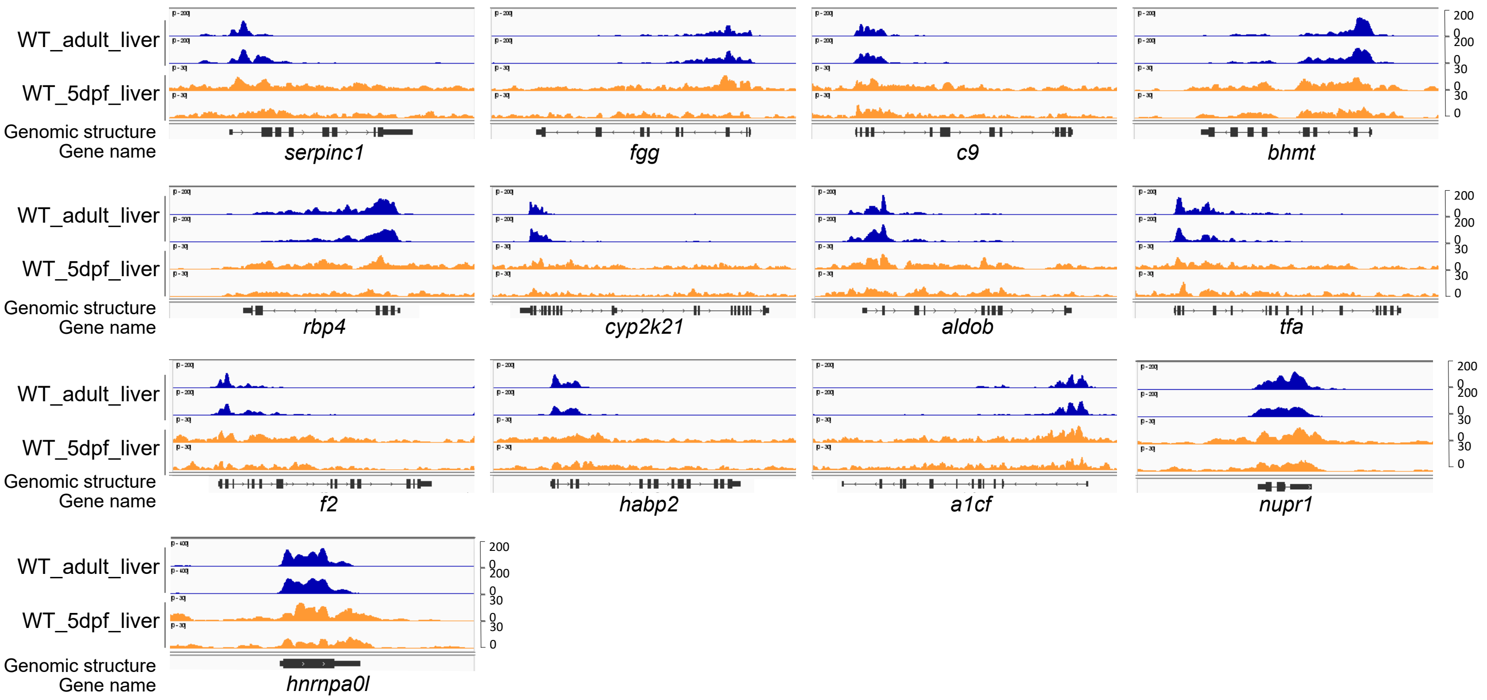
WT_adult_liver
WT_5dpf_liver
Genomic structure
Gene name
serpinc1
fgg
c9
bhmt
rbp4
cyp2k21
aldob
tfa
f2
habp2
a1cf
nupr1
hnrnpa0l
200
30
0

d

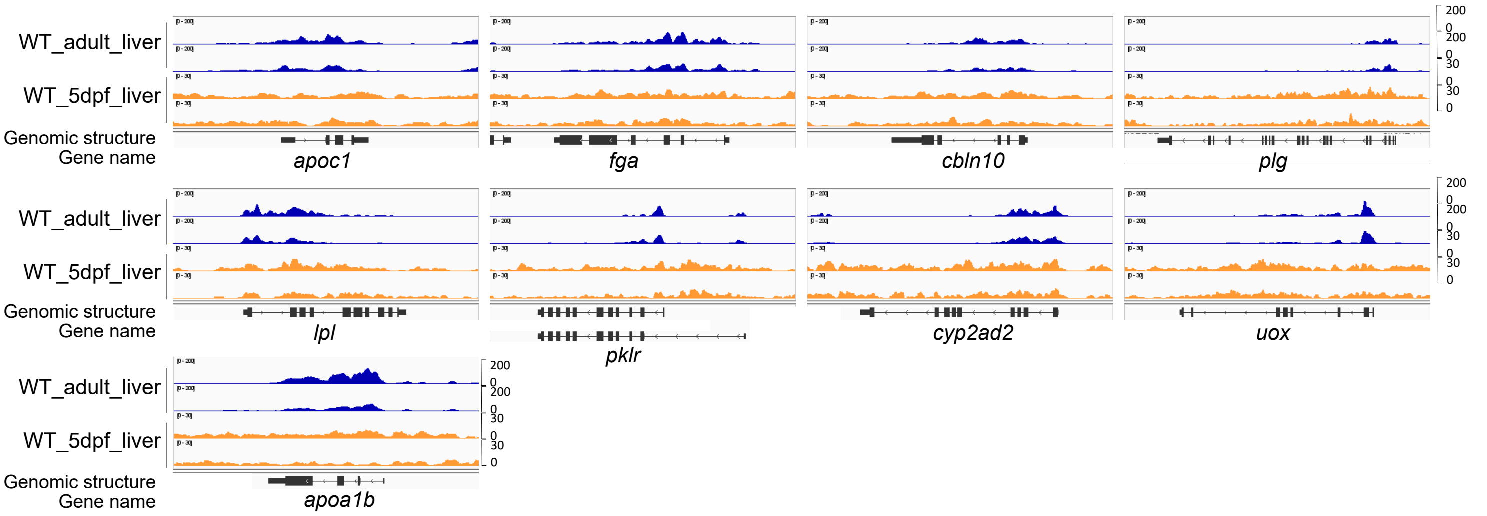
WT_adult_liver
WT_5dpf_liver
Genomic structure
Gene name
apoc1
fga
cbln10
plg
lpl
pklr
cyp2ad2
uox
apoa1b
200
30
0

e

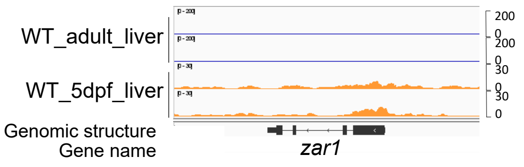
WT_adult_liver
WT_5dpf_liver
Genomic structure
Gene name
zar1
200
30
0

Fig. S9 Cross comparison of H3K4me3 enrichment in the genomic regions of 70 liver-enriched genes between WT adult and embryonic liver. a-d Five patterns were identified, including 27 genes showing relative TSS specific enrichment of H3K4me3 in both adult and embryonic liver (**a**), 20 genes showing relative TSS specific enrichment of H3K4me3 in the adult liver but not the embryonic liver (**b**), 13 genes showing a similar enrichment of H3K4me3 in the TTS together with gene body region in both adult and embryonic liver (**c**), 9 genes showing different patterns of H3K4me3 enrichment in the TSS together with gene body region (**d**) and one gene (*zar1*) showing the enrichment only in the embryonic liver but not in the adult liver (**e**).
